# Simulations of Long-Term Community Dynamics in Coral Reefs - How Perturbations Shape Trajectories

**DOI:** 10.1371/journal.pcbi.1002791

**Published:** 2012-11-29

**Authors:** Andreas Kubicek, Christopher Muhando, Hauke Reuter

**Affiliations:** 1 Leibniz Center for Tropical Marine Ecology, Bremen, Germany; 2 Institute for Marine Sciences of the University of Dar es Salaam, Zanzibar, Tanzania; CSIRO Marine Research, Australia

## Abstract

Tropical coral reefs feature extraordinary biodiversity and high productivity rates in oligotrophic waters. Due to increasing frequencies of perturbations – anthropogenic and natural – many reefs are under threat. Such perturbations often have devastating effects on these unique ecosystems and especially if they occur simultaneously and amplify each other's impact, they might trigger a phase shift and create irreversible conditions.

We developed a generic, spatially explicit, individual-based model in which competition drives the dynamics of a virtual benthic reef community – comprised of scleractinian corals and algae – under different environmental settings. Higher system properties, like population dynamics or community composition arise through self-organization as emergent properties. The model was parameterized for a typical coral reef site at Zanzibar, Tanzania and features coral bleaching and physical disturbance regimes as major sources of perturbations. Our results show that various types and modes (intensities and frequencies) of perturbations create diverse outcomes and that the switch from high diversity to single species dominance can be evoked by small changes in a key parameter.

Here we extend the understanding of coral reef resilience and the identification of key processes, drivers and respective thresholds, responsible for changes in local situations. One future goal is to provide a tool which may aid decision making processes in management of coral reefs.

## Author Summary

The degradation of coral reefs is a major threat for tropical coastal environments, worldwide. For this reason we developed a spatially explicit model which simulates competition in a benthic reef community under the influence of various environmental factors. Here we highlight the impact of two major perturbation types (mechanical disturbance events and temperature-induced bleaching events) on the long-term dynamics of a standard coral reef off Zanzibar Island, Tanzania. While mechanical disturbances are more non-specific and affect all organisms of the reef similarly, temperature-induced bleaching causes selective impact among coral species within the benthic community. Our results show clearly that complex systems which are organized of a multitude of diverse entities and hence feature complex emergent properties need to be analyzed on different integration levels rather than seen as a black box. Our tool may help to disentangle the combined effects of different perturbations and to analyze their respective impact on the benthic community of a coral reef. Hence, it will help to direct future research foci and to coordinate management measures for distinct site specific contexts.

## Introduction

Tropical coral reefs are highly productive but also fragile ecosystems that provide habitats for the coastal fauna and multiple services to local human communities [1]. Due to their high biodiversity, they exhibit a complex pattern of interactions between organisms and their environment with feedback loops within and between trophic as well as different hierarchical levels [2], and thereby facilitate a framework of non-linear dynamics which complicates a holistic analysis. Although extensive knowledge of corals, their responses to environmental change [3] and interaction with other organisms [4], and reef resilience [5] has been gained in the last few decades, the understanding of coral reef functioning is still far from being complete [6].

Reefs are increasingly under threat and many coral species are in danger of becoming extinct [7], due primarily to anthropogenic influence. Globally, coral reef systems are subject to rising sea surface temperatures which increase their susceptibility to bleaching, and to ocean acidification which erodes CaCO_2_ structures. Both stressors are chronically increasing and can be attributed to climate change [3], [5]. Additionally it is predicted that extreme weather events (e.g. el Niño or hurricanes) will strike with increasing frequency [8], [9]. Directly imposed human pressure upon coral reefs can have physical – e.g. by the use of destructive fishing techniques [10], [11], sedimentation [12], or anchorage [13]–[15] – or chemical – e.g. nutrients, sewage, pollution [11], [16] consequences.

The overall tendency of coral reef systems to react to changes in environmental conditions and anthropogenic influences can be described by the term resilience. It “… determines the persistence of relationships within a system […] and is a measure of the ability of these systems to absorb change […] and still persist.” [17]. In a coral reef it may be determined by species diversity, functional redundancy, life history of reef organisms, species functioning at different spatial and temporal scales, and connectivity to other reefs or habitat types [18], [19]. Reduced resilience can impose catastrophic regime shifts [20], [21] and in a reef often lead to a phase shift from coral dominated systems to alternative states; i.e. dominance of macroalgae [16],[22],[23] or of other benthic organisms [24], but see [25].

During the last two decades a series of ecological models have been applied to coral reef ecosystems. Among these we can find applications on various spatial and temporal scales. While Kleypas *et al.* [26] sought to approximate the possible geographic range for coral reefs to exist globally, other applications focus on conservation [27] or sustainable fishing regimes [28], [29]. There are yet other models at the regional, local and/or small scale [30]–[34] with the purpose to explore the influence of environmental conditions on spatial processes and interactions of coral reef community dynamics, and some of these models, like [35] are designed to aid management decisions.

Individual-based models (IBM) have proven to be an exceptional tool to tackle ecological questions with adequate detail [36]–[38] because properties of investigated ecological systems can be described very close to reality. By including, for example, heterogeneously varying individual interactions and spatial heterogeneity, IBMs considerably extend the range of ecological modelling [39]. In this study we focus on individual benthic organisms and their interaction with the environment because these processes and the spatial configuration of a community are the basis for environmental responses to perturbations in reality. There is a lot of knowledge on properties of individual coral colonies of various species; e.g. which symbionts they possess, how they grow, and how they react to thermal stress [40], [41], upon changing environmental settings in general, or if faced with other benthic organisms within their local neighborhood [42]–[45]. All of these factors are relevant for the understanding of coral reef functioning and should be included in an analysis of local reef dynamics.

To date, the application of individual-based models in the context of coral reefs has been somewhat limited, but interesting models have been developed for some investigations. Yniguez *et al.* [46] described the three-dimensional growth pattern of *Halimeda tuna*, a common macroalga in Florida Key reefs. Sleeman *et al.* [47] utilize an individual-based model to analyze different spatial arrangements of coral transplants in order to improve reef restoration measures. Koehl *et al.* [48] simulated larval transport in turbulent waters, and Brandt and McManus [49] investigated the spread of the white plague disease in various coral populations. Tam and Ang [50] present a strictly theoretical 3-dimensional model in which they describe disturbance-induced changes in a coral community with three different hypothetical coral growth patterns.

Here, we present a generic multi-species individual-based coral reef model in which scleractinian coral species and algae compete for space. This tool enables the analysis of key functions for coral reef resilience and the identification of major causes of phase shifts for local situations. In our example we apply a basic system with a standard parameterization for a typical Western Indian Ocean reef system.

In order to improve the understanding of how climate change and different modes of human interference affect the benthic composition of specific reef sites and their resilience we examine community responses under various environmental settings. Hence, we apply (1) different frequencies of temperature-induced bleaching, (2) two levels of mechanical disturbance regimes: a smaller one, which represents, for example, direct anchor damage or a smaller boat hitting the coral reef at low tide and a larger one representing, for example, damage by abrasion due to anchor chains, boat crashes or from fishing nets, and (3) both perturbations acting together to test the influences on the benthic community.

## Materials and Methods

### Model description

#### A) General

**Figure 1:**
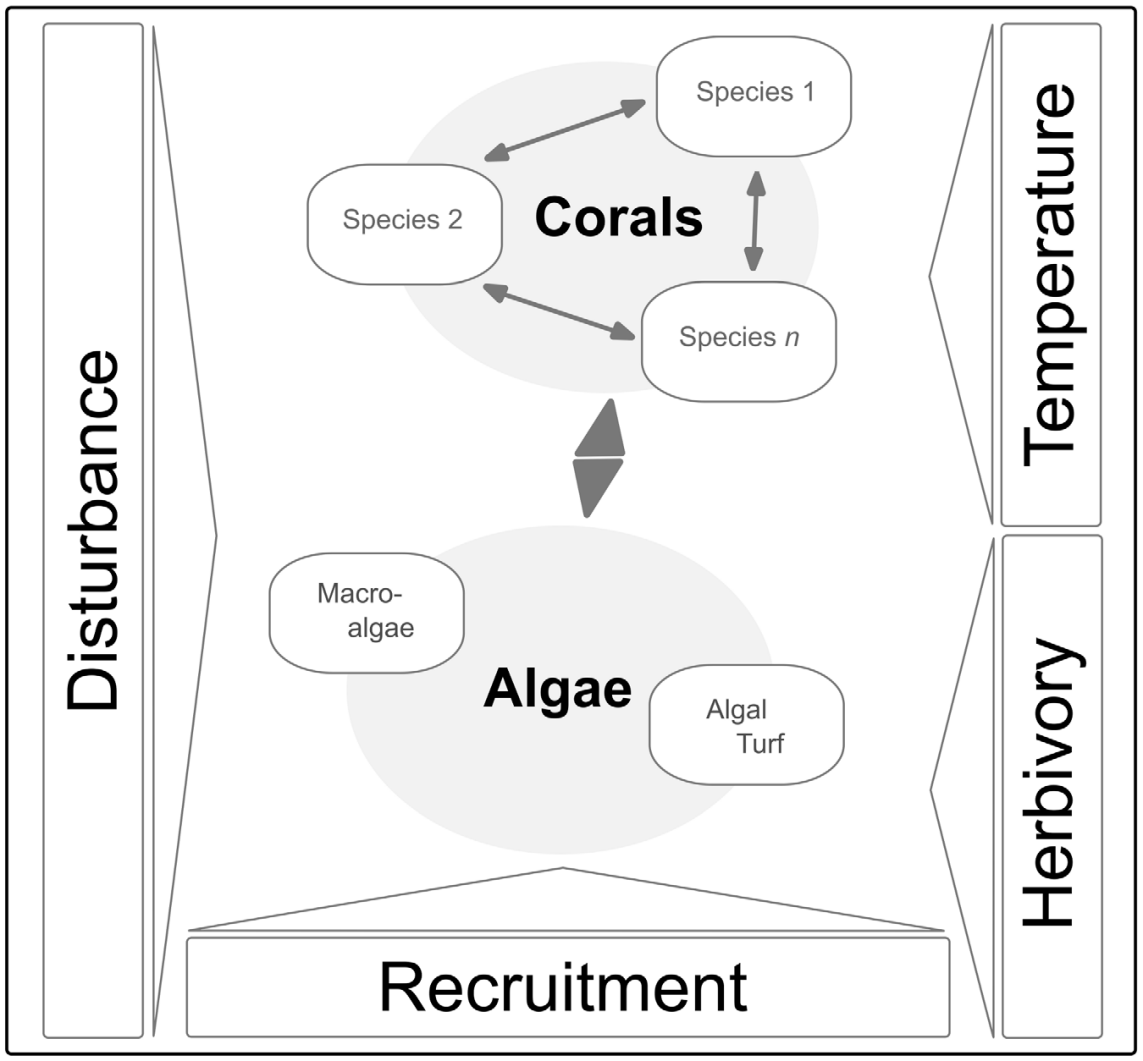
Overview of model components. Different coral species interact with each other and with two different types of algae. Mechanical disturbance affects the whole benthic community whereas high temperature triggers bleaching only in corals and herbivory affects only algae.

In this spatially explicit, individual-based model competition of benthic organisms (i.e. corals, macroalgae and turf) can be represented under various environmental settings (Fig. 1). The model consists of a two-dimensional continuous area on which all free space is considered as potential settling ground for all organism groups (see also Fig. S1). Individual life histories (e.g. recruitment, growth, death) of corals and algae and their interactions are described and each organism reacts specifically to environmental influences like temperature and mechanical disturbances (both investigated in detail). In the model, temperature influences the bleaching susceptibility of a coral colony and mechanical disturbance processes kill and remove all organisms inside the disturbed area. Macroalgal density is controlled by grazing by herbivores, which we implemented as a density dependent process. The model has been developed using the MASON multiagent simulation toolkit (see http://cs.gmu.edu/~eclab/projects/mason/) and is available at sourceforge (see http://sourceforge.net/projects/siccom). Details of the model implementation and parameterization will be described in the following sections.

#### B) Organisms

**Figure 2:**
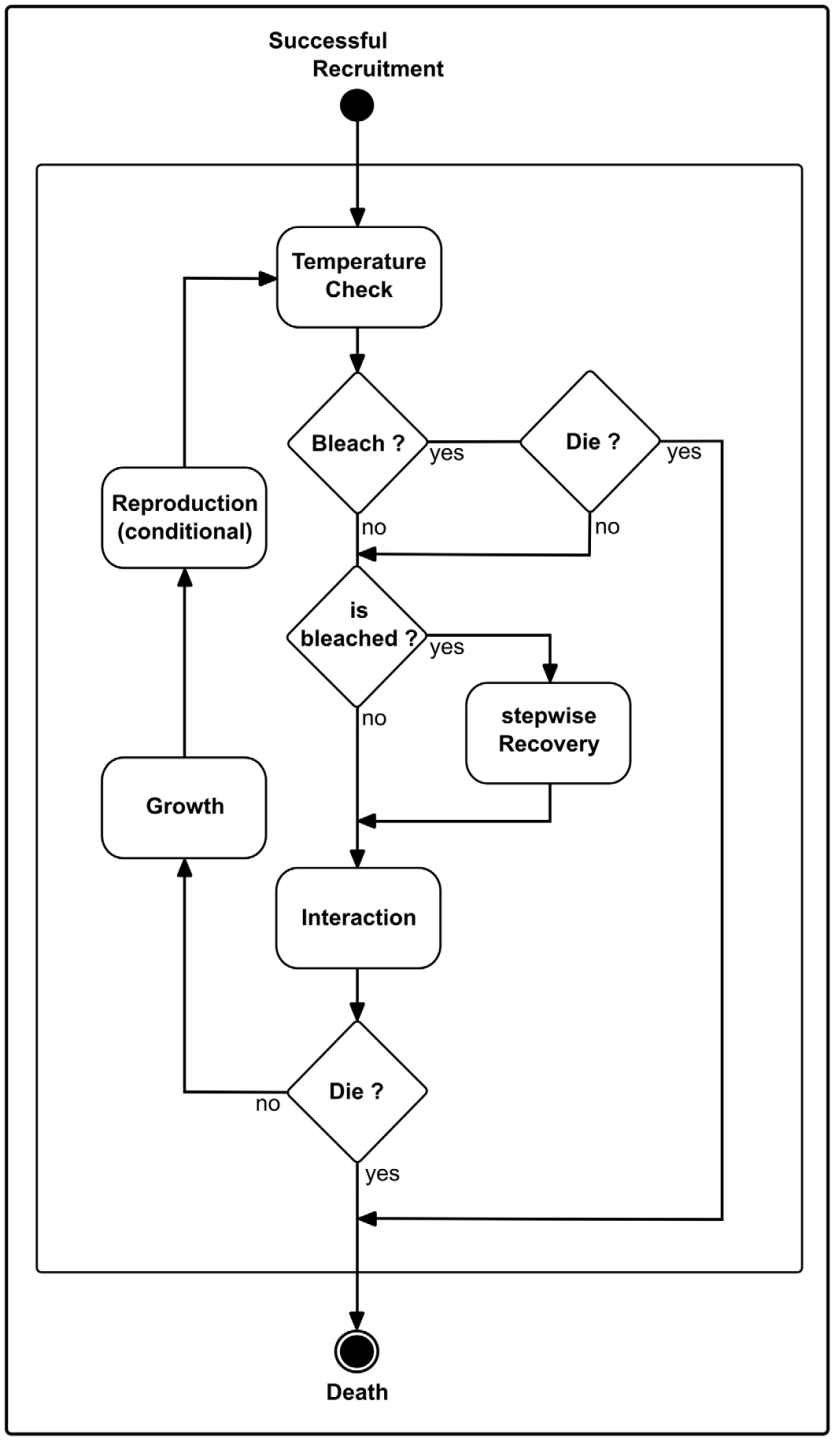
Flow chart of important processes of an individual coral colony. If recruit settling is successful, the coral colony enters its life-cycle. Within each iteration the temperature is checked, upon which it is decided if the colony will bleach or not. If it bleaches it can die or recover. In the next step the colony interacts with its neighborhood and if it does not die, it grows. Reproduction only takes place when the reproductive cycle allows it.

**Figure 3:**
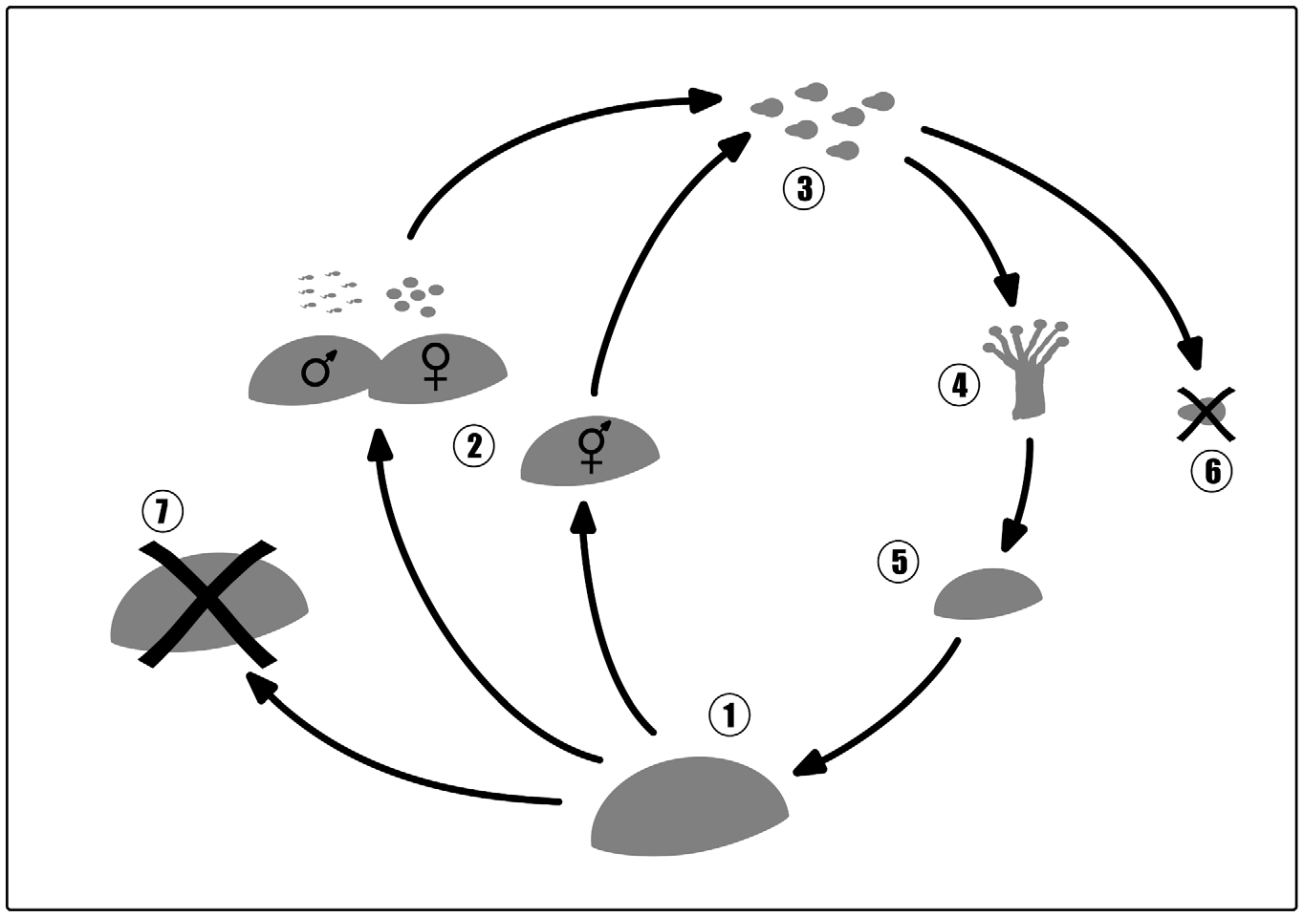
The life-cycle of a virtual coral which applies for massive and branching groups. 1) A mature coral colony produces gametes. 2) Hermaphroditic brooders directly release planula larvae, colonies of hermaphroditic broadcasters release bundles of eggs and sperm, and gonochoric broadcasting species release eggs or sperm, respectively. In the latter two modes fertilization takes place in the water column and planulae develop. 3) The larva is distributed and settles randomly on the simulation area. If it settles on another living organism (6) it will die and is removed. A larva that recruits on unoccupied space develops (4 and 5) into a new colony. 7) Overgrowth, disturbance or bleaching can lead to the death of a coral colony, which is then removed from the simulation.

Coral species are described with a detailed life history (Fig. 2) which may differ in growth pattern, growth rates, reproductive pattern, and susceptibility to temperature-induced bleaching. The life-cycle of a coral is simulated by considering all major processes; i.e. reproduction, release of gametes, and the settlement of recruits, as well as their growth or mortality due to external factors or interaction with neighbors (Fig. 3). The generic structure of species parameters (see Tables 1–3) allow the specification of a wide set of different functional coral groups.

**Table 1:** Linear extension rates of the implemented coral species.

Species	Mean Lateral Extension Rate (mm/year)	Location	Reference
*Porites lobata*	14.9	Ambon, Indonesia	[86]
	11.5	Cano Island, Costa Rica	[87]
	14.3	Java, Indonesia	[86]
	12.2	Lahaina, Maui, Hawaii	[88]
	11.0	Oahu, Hawaii	[89]
	6.4	Oahu, Hawaii	[90]
	7.8	Olosega, American Samoa	[91]
	14.7	Sulawesi, Indonesia	[87]
**All Average**	**11.6**		
*Porites lutea*	22.4	Abaiang Atoll, Kiribati	[92]
	5.7	Eilat, Gulf of Aqaba	[93]
	7.6	Enewetak, Marshall Islands	[94]
	19.4	Koh Phuket, South Thailand	[95]
	9.8	Kota Bontang, Indonesia	[96]
	11.0	Moorea, Society Islands	[97]
	16.7	Shikoku, Japan	[98]
**All Average**	**13.2**		
*Acrpora muricata*	123.3	Davies Reef, GBR	[99]
aka A.formosa	116.3	Hikkaduwa Nature Reserve, India	[100]
	62.6	Houtman Abrolhos, Western Australia	[101]
	34.2	Magnetic Island, Australia	[102]
	39.6	Magnetic Island, Australia	[103]
	86.4	Phuket, Thailand	[104]
**All Average**	**77.1**		
*Pocillopora damicornis*	32.2	Cano Island, Costa Rica	[86]
	50.2	Contadora Island, Panama	[105]
	25.0	Guam, Philippine Sea	[106]
	16.1	Lord Howe Island, GBR	[107]
	18.5	Oahu, Hawaii	[108]
	18.6	Oahu, Hawaii	[109]
	59.1	Pearl Islands, Panama	[110]
	18.0	Phuket, Thailand	[111]
	11.0	Rottnest Island, WA	[112]
	12.4	Solitary Islands, GBR	[86]
**All Average**	**26.1**		

**Table 2 pcbi-1002791-t002:** Reproduction parameters of the different coral species.

	Fix input (recruits cm^−2^ event^−1^)	No. of Recruitment Events (events year^−1^)	Spat size (mm)	Size at Maturity (diameter[cm])	Eggs/Larvae per cm^2^ surface area	Polyps per cm^2^ surface area	Eggs per polyp	Retention Factor	Surface Factor	Reproductive Mode
*Porites lobata*	0.01	1	1 [116]	8 [114]	1210 [113]	-	12 [119]	1.0E-8	1.5	gonochoric spawner [121]–[123]
*Porites lutea*	0.01	1	1 [116]	8 [114]	1375	19.1	72 [114]	1.0E-8	1	gonochoric spawner [121]–[123]
*Acropora muricata (formosa)*	0.01	1	1.2 [116]	4–7 [115]	109.5	15 [120]	7.2 [118]	4.5E-8	5	hermaphroditic spawner [121]–[123]
*Pocillopora damicornis*	0.01	12	2 [116]	4–7 [115]	2.5 [117]	-	-	1.5E-7	3	hermaphroditic brooder [124]

Values in parentheses indicate the respective references for an entry. All values without references are calculated from the other values.

**Table 3:** Miscellaneous parameters of the implemented coral species.

Species	Mean Lateral Extension Rate (mm/year)	Maximum Radius (cm)	Minimum Bleaching Temperature (°C)	Temperature where all corals bleach (°C)	Minimum Death Temperature (°C)	Temperature where all corals die (°C)
*Porites lobata*	11.6	300	29.9	31	29.4	32
*Porites lutea*	13.2	300	29.9	31	29.4	32
*Acropora muricata*	77.1	50	29.4	31	28.2	32
*Pocillopora damicornis*	26.1	30	30	31	21.5	30.4

The death temperature is calculated from above mentioned data to provide a continuous range upon which the probability for death at a specific temperature is determined once a coral is bleached.

We distinguish between massive and branching growth morphologies. Virtual corals basically grow with a constant rate extending their radius from a center, however, their growth performance is restricted by interaction with neighboring organisms or by their individual fitness (Fig. S2). Branching coral colonies are implemented as a 24-point star, of which the axis length represents the colony's extent in a given direction. Massive corals consist of the same ‘skeleton’ but here the endpoints of the star are connected to form a polygonal shape. The average radius of a colony is used to calculate the colony's cover and hemispherical surface area. To minimize edge effects, a coral's axis that expands over the borders of the simulation area cannot grow larger than the average radius of its colony.

In the model, coral reproduction determines recruitment numbers and depends on the specific reproductive traits. We differentiate between gonochoric broadcasters, hermaphroditic broadcasters and hermaphroditic brooders.

Two different processes contribute to the total amount of recruits. Internal recruitment from the simulated reef itself and external recruitment from adjacent reef systems; internal supply is estimated via a stock-recruitment relationship where the larvae output per mature colony is calculated by multiplying the gametes or larvae per cm^2^ with the surface area of massive and branching colonies, respectively. To estimate the surface area of a colony we multiply the hemispherical surface area (based on the average radius) of a colony, by the specific surface factor (see Table 2). The ‘external supply’ is divided into a basic rate (a fixed number of entering recruits per m^2^ per recruitment event) and a variable amount, which is defined as a multiple of the internal stock-recruitment. Thus, the focal reef patch can be considered statistically representative of the overall local situation and with a complementary connectivity factor that correlated to the distance to adjacent reef systems; i.e. a low value indicates low connectivity and a high value high connectivity. The amount of total recruit input for a species is then summed up from internal and external supplies and multiplied by a retention factor that integrates several factors, like (a) the reproductive mode, (b) the proportion of fertilized eggs, (c) predation, (d) the proportion of retained larvae and (e) early stage mortality. Settling larvae are distributed randomly on the simulation area. This approach allows for the depiction of a feedback process between the population density of a specific coral species and its number of recruits, while simultaneously considering the relation to neighboring reefs.

Currently effects of temperature are restricted to the induction of bleaching events. A model coral bleaches when a specific minimum bleaching temperature threshold is exceeded (see also parameterization: Temperature; Table 3: values according to [51], Fig. 3, mortality % transformed to 30–100% bleaching probability). In the case of bleaching, mortality occurs with a specified probability. Bleached corals that do not die will recover within the next 6 months with a reduced performance in growth and interaction strength during that phase. All affected rates (e.g. growth) increase linearly from 0% (full effect) to 100% (no effect) over this time span. Recruits settling on a bleached coral undergo a reciprocal survival probability, from 100% on a fully bleached coral to 0% on fully recovered individuals.

A coral colony can only die from a disturbance event, due to bleaching, or as a result of competitive interaction. Due to their hydrodynamic properties and the relatively small base of the colony, branching corals can break off if they are not sufficiently sheltered from surge and wave action. Virtual branching corals therefore are removed from the system with a 0.5% chance if they are not surrounded by two or more neighbors of at least the same size.

The life-cycle of macroalgae, and hence algal patch dynamics, take place in much shorter time-spans than that of scleractinian corals as algae grow faster and are generally subject to a higher frequency of trophic interactions. In the model, algae are controlled by grazing where the intensity depends on algal density (see section ‘Herbivory’). A virtual macroalga grows at first equally in vertical and horizontal orientation. As soon as it reaches its maximum allowed diameter, it only grows in height. The calculated diameter represents the alga's zone of influence towards other organisms, which also accounts for shading and abrasion in an area, larger than its actual diameter. In contrast to corals, algae can die from old age and are removed from the simulation after reaching the maximum age.

Algae also disperse faster than corals. We simulate algal dispersal in two distinct processes: yearly recruitment and fragmenting as soon as a threshold height is exceeded. For each fragment produced a certain value is subtracted from its height. The fragment can then settle within the vicinity of the mother plant or is lost if it leaves the simulation area. To avoid edge effects (lower algal densities near simulation borders) we created a margin around the actual simulation area in which algae can reproduce and grow, but which is not used for any calculation (see boundary conditions).

All filamentous and encrusting algae are combined into turf which we simulate as a grid with square cells (1×1 meters). In the model, we currently consider turf as a component that hampers coral recruitment by occupying potential settling ground (see also Interactions). The implemented life history traits are simple. Rather than taking account of recruitment or mortality of these plants with a very high turn-over, the relative cover of all turf algae within one grid cell is taken as measure of their density. It increases by 20% percent per month, or can be reduced by 50% due to herbivory which is implemented as a stochastic process depending on herbivore density.

#### C) Interactions

In the model, we put special emphasis on spatial interactions between different benthic organisms. Within an empty neighborhood corals grow unconstrained by interactions and expand into unoccupied space as long as they do not reach their maximum size. When a coral grows in the direction of another organism, its growth rate is reduced according to specified rules (Table 4) at the beginning of each time-step. Empirical studies revealed that some coral species are in principle competitively superior to others, i.e. if equal sized fragments of two species were placed in direct contact, one species overgrew the other one in most cases [52]. For this reason we applied a competition index, which together with the size of a colony determines the outcome of each competitive interaction. In all other cases, competitive success between members of the same growth morphology is only decided based upon individual size, location, and growth rate. We assume a slight competitive advantage for massive corals because they exhibit a more robust structure.

**Table 4:** The effect of interaction on growth of individual colonies or organisms.

		Competitor (C)		
		Massive Coral	Branching Coral	Macroalga
	**Massive Coral**	if F>C	if F>C	if F>C
		→ grows 10% less	→ no effect	→ no effect
				
		if F<C	if F<C	if F<C
		→ stops growing	→ grows 70% less	→ grows 30% less
**Focal**				
**Individual**	**Branching Coral**	F stops growing	if F>C	if F>C
**(F)**			→grows 30% less	→no effect
			if F<C	if F<C
			→ grows 70% less	→ grows 30% less
				
	**Macroalga**	→ no effect on	→ no effect on	→ no effect on
		growth	growth	growth

Focal individuals (F) are listed in the rows and their respective competitor (C) in the columns. As no literature values were available we made plausible assumptions for reactions on direct contact of corals.

A coral colony dies if more than a threshold amount (50% for branching colonies and 75% for massive colonies) of its size is overgrown by another organism. A coral recruit dies if it settles on space which is already occupied by another organism. If it settles on turf, the mortality probability is reciprocal to the percentage cover of the turf algae. Macroalgae can overgrow smaller coral colonies and can be overgrown by larger colonies, whereupon the competitively inferior individual is removed from the simulation. Inter-specific competition evokes death of a smaller alga if 50% of space is shared with a larger conspecific.

#### D) Herbivory

We assume that the simulated reef patch is within a coral reef network with low fishing impact and free access for herbivores. In the current version we represent herbivory as a simple process that controls algal population densities, where grazing rates are determined with a basic logistic function [53], [54]:
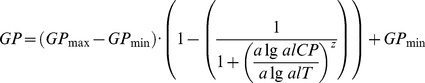
(1)where:

*GP* Grazing Probability

*GP_max_* maximal Grazing Probability

*GP_min_* minimal Grazing Probability

*algalT* Critical Threshold of algal cover percent

*z* Slope of the reaction

*algalCP* Algal Cover (Percent density)

### Parameterization

#### Standard parameterization

In the model, we represent typical attributes of a coral reef system around Unguja, the main island of the Zanzibar Archipelago, Tanzania (6.18928° S, 39.34137° W) which is one important coral reef location within the Western Indian Ocean.

We chose four typical scleractinian coral species for Zanzibar reef sites that represent important functional groups. *Porites lobata* and *Porites lutea* exhibit a massive colony shape while *Acropora muricata* (formerly aka *A. formosa* – see [55]) and *Pocillopora damicornis* feature branching growth patterns.

As no data on growth rates were available from Zanzibar, we applied average growth rates from literature (Table 1). *A. muricata* can reach a maximum radius of 50 cm and *P. damicornis* of 30 cm [56]. No data on the maximum size of massive corals was available, nevertheless we set a radius limit to 3 m, because larger colonies of these species are rarely observed in reefs around Zanzibar.

The main reproductive traits are derived from a literature search and were calculated from other variables if no direct data entry was found for a certain species (see also Table 2).

Rinkevich and Sakai [52] found that *P. lutea* is competitively inferior to *P. lobata* if fragments of approximately the same size are located next to each other. Therefore we applied the rule that *P. lobata* has a higher competition index, and can still grow with 30% of its input growth rate if it touches a larger *P. lutea* colony whose diameter is less than 25% bigger than its own.

Macroalgae which are mainly parametrized with data from *Sargassum ilicifolium* [57], [58] grow 30 cm per month, and can reach a maximum height of 60 cm and a maximum diameter of 45 cm. To account for the process of fragmenting we assumed that macroalgae fragment from a threshold height of 30 cm (a size at which surge can impact the alga) and for each produced fragment, their height is reduced by 5 cm. Algae recruit with a constant rate of 0.5 recruits m^−2^ once a year. The model performance with this parameterization was tested and evaluated with specialists from the Institute for Marine Sciences, Zanzibar prior to the experiments and the dynamics of virtual algae confirmed algal dynamics in Zanzibar reefs.

The temperature time series (1997–2010) used in this study originates from Chumbe Island, Zanzibar ([59], Muhando unpublished data). This time series included the 1998 El Niño mass bleaching event which caused severe coral bleaching and mortality; at this time, in March 1998 we measured temperatures approx. 2°C above average. To simulate longer time spans, single years of this data set were concatenated in a random order. As the 1998 temperature data trigger major bleaching events, the frequency of this year's occurrence can be set in the parameter list to simulate different scenarios of temperature extremes. To determine the strength of bleaching events sequential cumulative temperatures were calculated over periods of 20 days. For each coral species the highest temperature sum in each month was compared to the respective bleaching threshold (Table 3) and used for determining the bleaching probability. For the parameterization of bleaching we used data from McClanahan [51] and adjusted our model to produce similar bleaching and mortality results as occurred when temperature reached levels of the hottest month of 1998 in East Africa. In order to gain a basis for calculation of a dynamic bleaching reaction and in accordance with field observations we decided on a maximum temperature value at which all corals of a respective species bleach. From the onset of bleaching and the maximum value we were then able to derive continuous specific bleaching probabilities. The same method was applied to assess specific coral mortality rates (see Table 3).

Disturbance has strictly mechanical effects in the model and kills every organism within the affected area. Together with destructive fishing techniques [60]–[62], anchorage and boat contact have proven to have major destructive effects on scleractinian corals [13]–[15]. As mentioned above we chose two disturbance intensities; a smaller one (2–4 m in diameter) and a larger one (5–10 m in diameter). Both of the implemented intensity levels can occur alone or together within a simulation run and respective frequencies can be set for different scenarios.

In general, algal densities in Zanzibar coral reefs are still very low. Therefore we set the algal density threshold (*algalT* in equation (1)) to 5% cover. Herbivores are able to maintain this threshold and the initial probability of being grazed within one time step is set to 0.25 and can vary in the range between 0.2–0.3. The slope of the reaction is set to 2.0. The actual algal cover is then used to determine the grazing intensity or grazing probability for the next time step. The respective values were set and tested together with experts from the Institute for Marine Sciences, Zanzibar to best represent macroalgal dynamics around Zanzibar (Muhando unpublished data).

For the simulations, we defined a continuous area of 40×40 m on which we allow settlement of each organism group on unoccupied space. In order to minimize boundary effects we allowed macroalgal fragmenting to take place outside the actual simulation area within an additional 10 m margin that surrounds the field. Mechanical disturbance events were dealt with in an analogous way. The center of a disturbance is chosen randomly. In order to minimize edge effects (all individuals should have the same probability of being affected), the relevant area to determine the center of a disturbance extends the simulation area by the radius of the disturbance. Thus probabilities within the evaluated simulation area are equally distributed.

#### Environmental settings and scenario conditions

In the standard parameterization (Tables 2–3) a major bleaching event occurs every 15 years and small and large disturbance events every 12 and 60 months, respectively.

For the identification of long term community responses to particular environmental settings we ran several scenarios. Here we tested different frequencies of (1) extreme temperature events, occurring in 1 to 20 year intervals (2) two different intensities of mechanical disturbances (small: 2–4 m in diameter; large: 5–10 m in diameter) and (3) extreme temperature events under intermediate mechanical disturbance levels (small events every 12 and larger ones every 60 months) to assess the combined effect of the two perturbation types. All other parameters were kept constant. In the beginning of each simulation all coral species were distributed randomly over the simulation area with a respective coverage of 10%. Macroalgae were initiated with 5% coverage. Depending on the environmental settings it took about 50 to 100 years for the virtual reef to completely adapt to the specified condition. In this study we emphasized the influence of different perturbations on the long-term dynamics of a coral reef and do not intend to predict direct outcomes of pulse events. As the turnover rate and the pace of change in a reef happen comparably slow, an adequate time frame is needed to maintain stable population dynamics. Hence, to eliminate any influences of the initial distribution we analyzed only the last 500 years from each 1000 year scenario. The length of the simulations allowed for a reduction in the number of repetitions, because the stochastic occurrence of pulse events was thus largely compensated. The output from 10 repetitions was then combined for further analysis.

We concentrated our analysis on the dynamics of cover for each species. The total benthic cover is the sum of the relative cover of all species and as corals can overgrow each other the total sum may thus be >100%.

#### Sensitivity analysis and validation

In order to assess the sensitivity of the model to parameter changes and to ensure the reliability of the application we conducted a detailed validation and sensitivity analysis provided in Text S1. The outcomes of these analyses show how the model reproduces observed dynamics of typical reefs in Zanzibar (Figures S2, S3, S4 a and S4 b). The output is highly sensitive to alterations of the larvae retention factor (Fig. S5), which combines many assumptions and cannot be validated with empirical data. Varying growth rates (Fig. S6) and susceptibility to extreme temperatures (Fig. S7) only produce considerable change if qualitative properties between species are affected. Nevertheless, for the parameterization of particular reef sites these two parameters have to be handled with greatest care, and especially specific susceptibilities to temperature-induced bleaching has a large influence on the reef resilience, the resulting community composition and hence on the fate of a coral reef. The variation of parameters relevant for herbivory, do not evoke any noteworthy changes under the given configuration (Fig. S8).

## Results

### General pattern

At standard settings, the massive *P. lutea* dominated, followed by the branching *A. muricata*. The other two species both leveled at around 5% coverage. *A. muricata* exhibited the highest fluctuations in relative cover and *P. lobata* the lowest.

Surveyed exclusively, both perturbation modes triggered similar responses in the benthic community; not only changes in overall benthic coverage but also alterations in community composition. For low frequencies of rare events, massive growth forms clearly dominated the system with high total benthic cover. Macroalgal cover is negligibly low. If perturbations occurred at intermediate frequencies, overall cover decreased and space was nearly evenly distributed between the two different growth morphologies. At the highest perturbation levels, the total benthic cover was very low, the relative fraction of algae increased strongly and massive species were displaced by branching ones.

Nevertheless, single effect testing of major bleaching events and mechanical disturbances revealed that both perturbation types triggered differential responses of the benthic community. These are discussed further below.

### Bleaching

**Figure 4:**
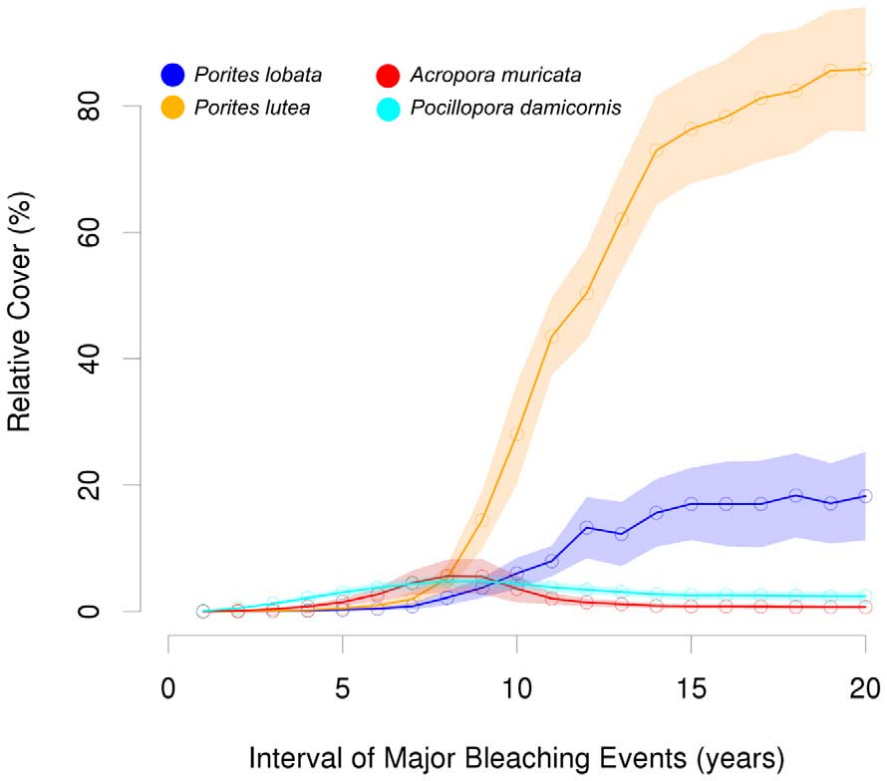
Mean relative cover (± SD) of the different coral species in relation to varying frequencies of major bleaching events without the influence of physical disturbances. A clear shift in coral community structure can be observed when bleaching frequencies increase from 8–9 years.

Without the influence of mechanical disturbances, there was nearly no visible change in the total benthic cover (which was always ≥99%), if major bleaching events occurred in intervals of 16 years or higher. The community was dominated by *P. lutea* with a relative coverage of ∼80% or larger (Fig. 4), followed by *P. lobata* (∼18%). When extreme temperatures occurred every 10 to 15 years, major changes in relative cover arose mainly for *P. lutea* which decreased from 76.1 to 28.1% coverage, and *P. lobata* which decreased from 17.0 to 6.0% coverage. For the dominance of *P. lutea* we identified a threshold of 8–9 years between major bleaching events (Fig. 4).

At very high bleaching frequencies the total coral cover did not exceed 2% coverage and macroalgae dominated the benthic community. Branching corals dominated if bleaching events occurred every 8 years or more often. Within this range the cover of *P. damicornis* increased gradually and *A. muricata* only dominated if extreme temperature events happened between 7 and 9 year intervals.

### Disturbance

**Figure 5:**
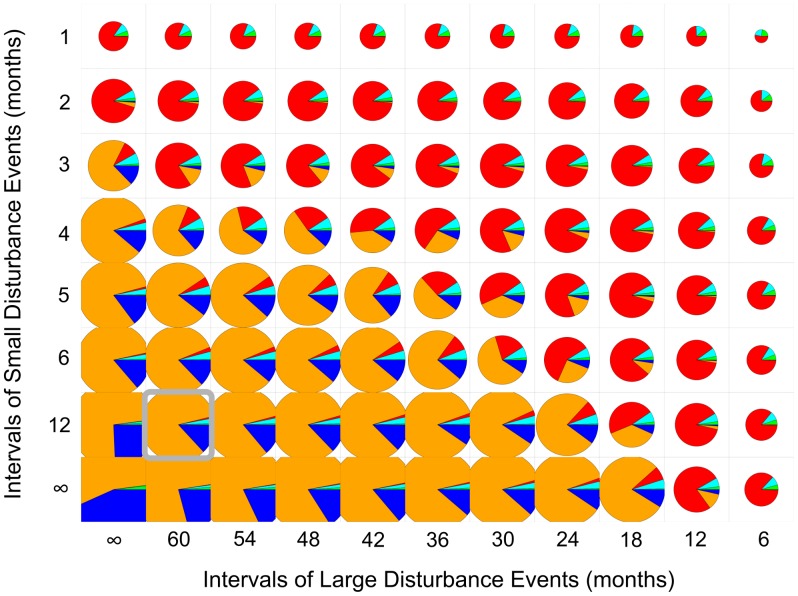
Effect of different frequencies of large (x-Axis) and small (y-Axis) disturbance events without the influence of major bleaching events. The diameter of a pie chart denotes the percentage of the total benthic cover. Standard settings of mechanical disturbances, with smaller intensities occurring every 12 and larger ones every 60 months, are indicated by the gray frame. The colors represent benthic organisms as follows: *P. lobata* in blue, *P. lutea* in orange, *A. muricata* in red, *P. damicronis* in cyan and macroalgae in green.

Without the influence of extreme temperature events both applied disturbance intensities triggered nearly the same community responses, although the frequency of the smaller size events had to be far higher for a similar effect (Fig. 5). Increasing disturbance frequencies abetted dominance shifts from massive to branching growth forms, resembling the pattern of the single effect of bleaching. The interface from the dominance of *P. lutea* to *A. muricata*, where the community featured the highest evenness, was restricted to a small range of configurations. At standard frequencies (i.e. for smaller intensities every 12, and for larger ones every 60 months) the total benthic cover was 117% (see section ‘Environmental settings and scenario conditions’) where *P. lutea* clearly dominated, followed by *P. lobata*, the two branching coral species covered together ∼3%, and macroalgae covered 0.5%. Under highest applied frequencies of mechanical disturbance events the total benthic cover amounted to 16.2%, and massive corals nearly disappeared altogether (<0.1% cover) with only the faster growing branching corals still present in the system.

### Combined effects of bleaching and disturbances

**Figure 6:**
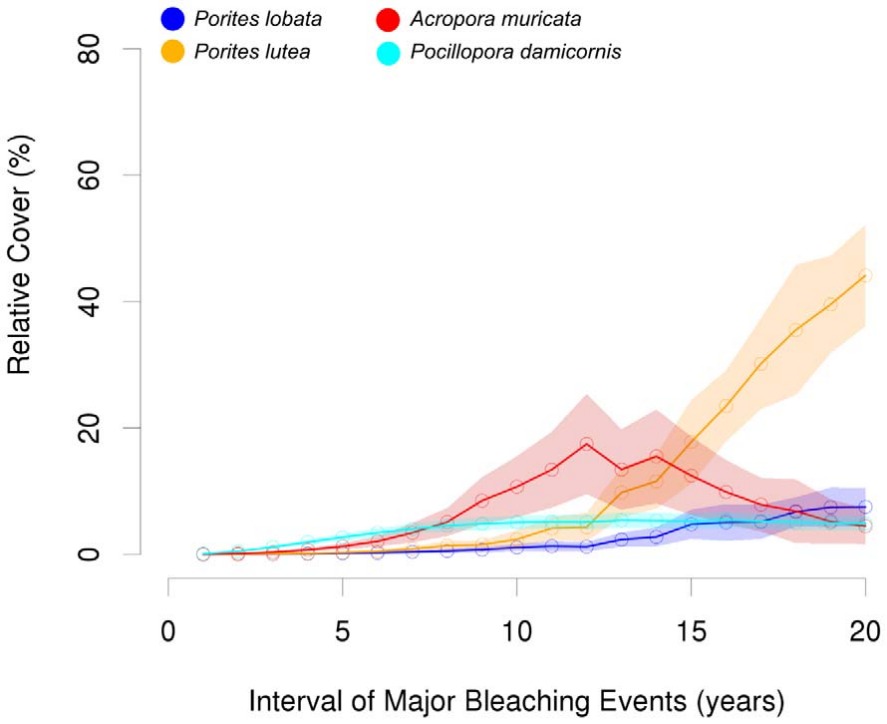
Mean relative cover (± SD) of the different coral species in relation to varying frequencies of major bleaching events at intermediate physical disturbance levels. A clear shift in coral community structure can be observed when bleaching frequencies increase from 10–15 years.

Under the regime of both applied perturbations, where different frequencies of bleaching events were tested under standard mechanical disturbance levels the effect of bleaching was amplified (Fig. 6). Similar to the single effect scenario the total coral cover was low and macroalgae dominated at very high frequencies of bleaching events. At 20 year intervals, the total benthic cover did not exceed 63%, and as in the assessment of the sole bleaching effect, *P. lutea* dominated the community, while all other species' coverage stayed below 10%. *P. lobata* did not exceed 10% cover within any tested frequency. Also contrasting is the behavior of *A. muricata*. It dominated the community at extreme temperature intervals between 8 and 14 years but its cover decreased tremendously at higher frequencies. *P. damicornis* again increased its relative cover gradually at high frequencies, but then stayed more or less constant at levels around 5% coverage if major bleaching occurred every 9 years or more seldom.

The ratio of massive and branching corals was nearly 4∶5 at 15 year intervals for major bleaching, and the dominance threshold for *P. lutea* was shifted from 8–9 year intervals up to 14–15 year intervals in the combined perturbations scenarios.

## Discussion

The model outcome reflects findings of empirical studies in many regards and provides interesting insights on the influence of multiple perturbations on coral reef communities. Massive corals are generally slow growing but exhibit a strong physical structure. Provided their tissue is healthy and the defense intact, they are quite resistant to overgrowth by other organisms, like branching coral species or macroalgae which competitively mainly rely on their faster growth rates [63]. In addition, both of the observed massive species feature low susceptibilities to bleaching among the tested corals, and a low bleaching-induced mortality. The combination of slow growth and high endurance implies small fluctuations in relative coverage. As yet another consequence of the above mentioned properties, massive species outcompete their benthic opponents gradually if perturbation levels are low, where *P. lutea* overrules *P. lobata* and dominates the community due to its higher growth rate (see Fig. 4 and Fig. 5 at low perturbation levels). This effect is also pronounced by the applied stock-recruitment relationship which leads to a self-enhancing process; i.e. individual colonies grow fast, reach maturity earlier, and produce many propagules again resulting in a higher recruitment rate and new colonies.

The branching species *A. muricata* exhibits the highest fluctuations in population size and relative coverage. It has the fastest growth rate of all simulated species and, given there is enough space, can dominate the benthic community within a few years due to the self-accelerating process produced by the stock-recruitment relationship described above. On the other hand, its bleaching vulnerability to thermal stress is the highest within the tested community, which leads to considerable losses due to extreme temperature events. *Acropora* and *Pocillopora* are genera with many species which feature high susceptibility to bleaching [64]. Accordingly, *A. muricata* is the only species in our model which shows bleaching responses in years when temperatures did not rise as high as in 1998. For *P. damicornis* the situation is different. In our model, this species is the least susceptible of all tested species in terms of bleaching, but when a colony bleaches it nearly always dies. Therefore, extreme weather events have nearly analogous effects on the mortality for both branching species. The long term survival of a species is hence strongly dependent on larval input from the outside and thence influenced by asynchronies on the regional and trans-regional scale.

The results show that different types and levels of perturbations can lead to very diverse community responses and thereby reef fate. In a mechanical disturbance event, the only reason for an individual to be affected is if it is located at the wrong spot at the wrong time. At higher frequencies of these events the growth rate and recolonization speed of an organism decides winners and losers. Extreme temperature events feature a more selective pattern. Populations of higher susceptibility to thermally induced bleaching suffer the highest losses. Their lower abundance evokes a reduced reproductive output for the next spawning event which, in the long run, might constitute a disadvantage over less susceptible species. Generally, mechanical disturbances occur locally and do not affect the regional coral populations, so that neighboring reefs can serve as a source for new recruits and the local population can recover comparatively fast. An increase of the sea surface temperature is affecting populations at a larger spatial extent and evokes a regional effect. Conspecifics of a sensitive species are affected similarly region-wide if they are not protected from the high temperatures in some way. In such a scenario the larval support between reefs can be hampered tremendously and the risk of extinction increases [65].

Structural complexity of a coral reef is a very sensitive emergent property and high coral cover does not thoroughly imply high ecosystem function (e.g. 3-dimensional framework) [66]. Perturbations at too high or too low frequencies cause a loss of biodiversity and thereby structural complexity. Very low frequencies and/or intensities of mechanical disturbance events mostly lead to dominance of species with high competitiveness or endurance and it is just a matter of time until individuals of these species overgrow and displace inferior organisms. In our example, massive corals dominate under very low disturbance regimes. They do not provide as much structure as branching corals and so structural complexity is lost which might cause unfavorable conditions for other reef associated organisms which are deprived of hiding places. One example is Changuu reef, close to the city of Zanzibar, a very exploited site by the means of fishing and under strong influence of waste water, and hence pollutants and nutrients from the town [67]–[69]. Muhando et al. [70] found that corallimorpharians covered 14% of the reef, mainly on the reef crest and flat. Here a large part of the (still persisting) coral cover is made up by *Galaxea astreata* and *Porites rus* [71], both of which seem to be quite resistant to environmental change and possess strong competitive traits over other taxa (e.g. corallimorpharians) but which facilitate scarce structure. Therefore this site exhibits low biodiversity of fish and other associated organisms.

Under very high physical disturbance levels the overall coral cover is generally very low. These conditions are detrimental for massive corals and thus provide free settling ground for branching species . On the other hand branching corals are affected as well, because without shelter from surge or strong currents, they might break off. Nevertheless, due to their high growth rates they play a crucial role in post-disturbance recolonization [72] and reef building event though recovery periods may be too short for slower growing massive coral species. Although branching coral species are displaced by massive species in Indo-Pacific reef sites [73], this trend cannot only be attributed to the two perturbation types, addressed in this study, but is also owed to the fact that massive species are more resistant to other threats, like pollution [74] or more selective mechanical disturbances like hurricanes, which invoke strong surge and currents [72].

In the given context and despite the limited number of represented species, the principles of the intermediate disturbance hypothesis [75] are well resembled by our model results and in line with previous studies [30], [50]. At high and low disturbance regimes one species dominates, whereas the evenness is highest at intermediate disturbance levels.

In order to investigate the sole effects of perturbations on long term dynamics of a reef without the effect of extinctions, we made two major assumptions. First, we kept the grazing intensity on algae more or less constant, assuming that the herbivore community is completely independent of the structural complexity that the reef is providing. The general view is that the abundance of herbivorous fish and other associated reef organisms is directly influenced by the availability of sheltering places provided by the reef structure [19], [76], [77], and especially by the abundance of branching corals [66]. A loss of a reef's structural complexity therefore reduces herbivore abundance, macroalgae are not grazed efficiently and their populations can proliferate freely. Resulting stands of macroalgae decrease coral recruitment success [78], [79] which can then produce a feedback in which macroalgae may take over a once coral dominated system.

Secondly, we assume a constant larval supply from the outside, on top of the applied stock-recruitment relationship. According to McClanahan *et al.* [80] the extinction risk of all species tested here as a response to bleaching seems to be very low in the Western Indian Ocean region, with *Pocillopora* and *Acropora* showing a probability of 12 and 11%, respectively, and the massive *Porites* of 7%. In contrast to the observations that McClanahan *et al.* [81] made in Kenya, where they observed that *Pocillopora* was completely depleted at protected sites and close to gone from unprotected ones, *P. damicornis* survives in our simulations. This is due to our second assumption that nearby source reefs were not as much affected by high temperature influences as the focal reef, either because they are located in deeper waters where the heating effect is alleviated or by mixing of water masses.

These two assumptions implicate limitations for model extrapolations. To transfer the model to other sites, specific adaptations to the local conditions and to local species parameters have to be made. This also applies to the other explicit and implicit assumptions, underlying the model development, such as the 2-dimensional spatial configuration, the choice of reef components, and their rules of interaction and competition. Additionally, the applied parameter specifications, most of all the relative rates of growth, the reaction to bleaching and larval recruitment, are important for the model outcome.

Although there are many types of perturbations, in this article we concentrated on two of the main ones, which concern coral reefs and most probably will have increasing impact in the future; bleaching as a result of global climate change and anchor and boat damage due to an increased demand for food and in consequence more fishing on reefs. The framework of our model allows for the addition of other substantial threats, like ocean acidification, nutrient input (which hampers coral fitness but may have a positive effect on algae), coral diseases, and sedimentation, all of which will affect resource allocation in virtual corals and thereby decrease growth rates and/or competitive strength. Intensive fisheries constitute a fundamental problem for future coral reefs which should be treated with special attention. Enhanced fishing pressure depletes stocks, which most probably results in either higher effort (i.e. more fishing trips) and/or the utilization of more efficient fishing gear that often has destructive capacities. Both practices increase the risk of reef degradation (i.e. by more frequent anchorage and boat damage, or directly through fishing gear). In the longer run this might evoke a downward spiral.

While we are still far away from the point in which we can represent all features of these highly complex biota, our study extends model capabilities from former coral reef models. This extension improves the accuracy of involved processes as well as on the spatial scale and resolution of the simulated reef system. The model reflects important coral reef dynamics and allows us to test different scenarios relevant for resilience research. Its generic and modular structure and the potential to parameterize different reef components and different coral species, as well as environmental influences provides the possibility for adapting it to represent many reef sites worldwide.

The model clearly shows that even when perturbation regimes are kept constant the system never reaches a stable state and stochasticity produces a continuously adapting and fluctuating community response. Pulse events can have a strong influence on ecosystem functioning, especially if they amplify each other's effects or stress the ecosystem in addition to a prevailing chronic disturbance [21] such as increasing nutrient loads or sedimentation. This may cause a shift of general system properties, and lead to, for example, a coral-algae phase shift [82].

Several studies that have quantified resilience with a small number of key parameters have already provided interesting insights [83]–[85]. Nevertheless, measuring resilience in such complex systems as coral reefs is hard to accomplish because the high number of site specific relations and components complicates generalization and extrapolation of specific findings considerably. However, management has a high interest in, and demand for, tools which aid in tracing and identifying distinct drivers for the decline of specific coral reefs. Only a clear recognition of drivers, mechanisms and causes makes it possible to establish or introduce adequate protection measures. Generic frameworks, like the model presented here, allow a fairly easy integration of site specific features and may serve as an appropriate basis for management support tools.

## Supporting Information

Figure S1 **Screen shot of the graphical user interface during a simulation.** Colonies of the two massive species *P. lobata* (blue polygons) and *P. lutea* (orange polygons), and the two branching species *A. muricata* (red stars) and *P. damicornis* (cyan stars) compete for space with each other as well as with macroalgae (green dots) and algal turf (green squares). The free space which is largely covered by macroalgae and turf indicates post-disturbed areas.
(TIF)

Figure S2 **Interaction between different neighboring coral colonies.** Different growth forms interact in different ways which is shown for (a) branching colonies, (b) massive with branching colonies and (c) massive colonies, respectively. Growth is clearly restricted in direction of neighboring individuals and thus the common irregular shapes arise.
(TIF)

Figure S3 **The population's growth performance of** ***Porites lutea*** **under different disturbance levels and crowding regimes.** high disturbance levels, which imply low crowding (left), and low disturbance levels which imply high crowding (right), respectively.
(TIF)

Figure S4 **Validation of the community response to a major bleaching event.** In (a) the relative benthic cover of Chumbe Island MPA is shown before and after the 1998 bleaching event (adapted from Muthiga *et al.* [7] in Text S1). Chart (b) shows a time line of the model output that represents the impact of the 1998 bleaching event.
(TIF)

Figure S5 **Retention factors of all coral species were varied by ±10% of their standard value and respective community responses plotted for each setting.** Vertically the retention rates of the two massive species (*P. lobata* in blue and *P. lutea* in orange) are varied and horizontally the ones of the two branching species (*A. muricata* in red and *P. damicornis* in cyan). The size of the pie chart indicates the total benthic cover and the gray box indicates the standard values.
(TIF)

Figure S6 **Growth rates of all coral species were varied by ±10% of their standard value and respective community responses plotted for each setting.** Vertically the growth rates of the two massive species (*P. lobata* in blue and *P. lutea* in orange) are varied and horizontally the ones of the two branching species (*A. muricata* in red and *P. damicornis* in cyan). The gray box indicates the standard values.
(TIF)

Figure S7 **The minimum bleaching temperature for each coral species was varied by ±0.4°C of its respective standard value and the community response was plotted for each setting.** Vertically the growth rates of the two massive species (*P. lobata* in blue and *P. lutea* in orange) are varied and horizontally the ones of the two branching species (*A. muricata* in red and *P. damicornis* in cyan). The size of the pie chart indicates the total benthic cover and the gray box indicates the standard values.
(TIF)

Figure S8 **Variation of key parameters for herbivory (±10%) shows no decisive effect on coverage and community composition.** The colours represent benthic organisms as follows: *P. lobata* in blue, *P. lutea* in orange, *A.muricata* in red, *P. damicronis* in cyan and macroalgae in green.
(TIF)

Text S1 **Appendix with a detailed sensitivity analysis and a hierarchically structured validation for the model.**
(PDF)

## References

[1] Moberg F, Folke C (1999) Ecological goods and services of coral reef ecosystems. Ecol Econ 29: 215–233.

[2] Hatcher BG (1997) Coral reef ecosystems: how much greater is the whole than the sum of the parts? Coral Reefs 16: 77–91.

[3] Hoegh-Guldberg O, Mumby PJ, Hooten J, Steneck RS, Greenfield P, et al (2007) Coral Reefs Under Rapid Climate Change and Ocean Acidification. Science 318: 1737–1742. 18079392 10.1126/science.1152509

[4] Birrell CL, McCook LJ, Willis BL, Diaz-Pullido GA (2008) Effects of benthic algae on the replenishment of corals and the implications for the resilience of coral reefs. Oceanogr Mar Biol Annu Rev 46: 25–63.

[5] Hughes TP, Baird AH, Bellwood DR, Card M, Connolly SR, et al (2003) Climate Change, Human Impacts, and the Resilience of Coral Reefs. Science 301: 929–933. 12920289 10.1126/science.1085046

[6] Hughes TP, Bellwood DR, Folke C, Steneck RS, Wilson J (2005) New paradigms for supporting the resilience of marine ecosystems. Trends Ecol Evol 20: 280–286. 10.1016/j.tree.2005.03.022 16701400

[7] Carpenter KE, Abrar M, Aeby G, Aronson RB, Banks S, et al (2008) One-Third of Reef-Building Corals Face Elevated Extinction Risk from Climate Change and Local Impacts. Science 321: 560–563 doi:10.1126/science.1159196. 18653892 10.1126/science.1159196

[8] Timmermann A, Oberhuber J, Bacher A, Esch M, Latif M, et al (1999) Increased El Nino frequency in a climate model forced by future greenhouse warming. Nature 398: 1996–1999.

[9] Harley CDG, Hughes AR, Hultgren KM, Mine BG, Sorte CJB, et al (2006) The impacts of climate change in coastal marine systems. Ecol Lett 9: 228–241. 16958887 10.1111/j.1461-0248.2005.00871.x

[10] McManus JW, Reyes RB, Nanola C (1997) Effects of Some Destructive Fishing Methods on Coral Cover and Potential Rates of Recovery. Environ Manage 21: 69–78. 8939786 10.1007/s002679900006

[11] Edinger EN, Jompa J, Limmon GV, Widjatmoko W, Risk MJ (1998) Reef Degradation and Coral Biodiversity in Indonesia. Effects of Land-based Pollution, Destructive Fishing Practices and Changes Over Time. Mar Pollut Bull 36: 617–630.

[12] Muzuka ANN, Dubi AM, Muhando CA, Shaghude YW (2010) Impact of hydrographic parameters and seasonal variation in sediment fluxes on coral status at Chumbe and Bawe reefs, Zanzibar, Tanzania. Estuar Coast Shelf Sci 89: 137–144.

[13] Davis GE (1977) Anchor Damage to a Coral Reef on the Coast of Florida. Biol Conserv 11: 29–34.

[14] Rogers CS, Garrison VH (2001) Ten Years after the Crime: Lasting Effects of Damage from a Cruise Ship Anchor on a Coral Reef in St. John. Bull Mar Sci 69: 793–803.

[15] Dinsdale EA, Harriott VJ (2004) Assessing anchor damage on coral reefs: a case study in selection of environmental indicators. Environ Manage 33: 126–139. 14625652 10.1007/s00267-003-3056-9

[16] Szmant AM (2002) Nutrient Enrichment on Coral Reefs: Is It a Major Cause of Coral Reef Decline. Estuaries 25: 743–766.

[17] Holling CS (1973) Resilience and Stability of ecological Systems. Annu Rev Ecol Evol Syst 4: 1–23.

[18] Nyström M, Folke C (2001) Spatial Resilience of Coral Reefs. Ecosystems 4: 406–417.

[19] Nyström M, Graham NAJ, Lokrantz J, Norström AV (2008) Capturing the cornerstones of coral reef resilience: linking theory to practice. Coral Reefs 27: 795–809.

[20] Scheffer M, Carpenter S, Foley JA, Fole C, Walker B (2001) Catastrophic shifts in ecosystems. Nature 413: 591–596. 11595939 10.1038/35098000

[21] Scheffer M, Carpenter SR (2003) Catastrophic regime shifts in ecosystems: linking theory to observation. Trends Ecol Evol 18: 648–656.

[22] McCook LJ (1999) Macroalgae, nutrients and phase shifts on coral reefs: scientific issues and management consequences for the Great Barrier Reef. Coral Reefs 18: 357–367.

[23] Nyström M, Folke C, Moberg F (2000) Coral reef disturbance and resilience in a human-dominated environment. Trends Ecol Evol 15: 413–417. 10998519 10.1016/s0169-5347(00)01948-0

[24] Norström A, Nyström M, Lokrantz J, Folke C (2009) Alternative states on coral reefs: beyond coral–macroalgal phase shifts. Mar Ecol Prog Ser 376: 295–306.

[25] Zychaluk K, Bruno JF, Clancy D, McClanahan TR, Spencer M (2012) Data-driven models for regional coral-reef dynamics. Ecol Lett 15: 151–158. 22188529 10.1111/j.1461-0248.2011.01720.x

[26] Kleypas JA, Buddemeier RW, Archer D, Gattuso J-P, Langdon C, et al (1999) Geochemical Consequences of Increased Atmospheric Carbon Dioxide on Coral Reefs. Science 284: 118–120 doi:10.1126/science.284.5411.118. 10102806 10.1126/science.284.5411.118

[27] Melbourne-Thomas J, Johnson CR, Perez P, Eustache J, Fulton EA, et al (2011) Coupling Biophysical and Socioeconomic Models for Coral Reef Systems in Quintana Roo, Mexican Caribbean. Ecol Soc 16: 23.

[28] McClanahan TR (1995) A coral reef ecosystem-fisheries model: impacts of fishing intensity and catch selection on reef structure and processes. Ecol Modell 80: 1–19.

[29] Gao L, Hailu A (2012) Ranking management strategies with complex outcomes: An AHP-fuzzy evaluation of recreational fishing using an integrated agent-based model of a coral reef ecosystem. Environmental Modelling & Software 31: 3–18.

[30] Langmead O, Sheppard C (2004) Coral reef community dynamics and disturbance: a simulation model. Ecol Modell 175: 271–290.

[31] Mumby PJ (2006) The impact of exploiting grazers (*Scaridae*) on the dynamics of Caribbean coral reefs. Ecol Appl 16: 747–769. 16711060 10.1890/1051-0761(2006)016[0747:tioegs]2.0.co;2

[32] Mumby PJ, Hastings A, Edwards HJ (2007) Thresholds and the resilience of Caribbean coral reefs. Nature 450: 98–101. 17972885 10.1038/nature06252

[33] Fung T, Seymour RM, Johnson CR (2011) Alternative stable states and phase shifts in coral reefs under anthropogenic stress. Ecology 92: 967–982. 21661558 10.1890/10-0378.1

[34] Buenau KE, Price NN, Nisbet RM (2012) Size dependence, facilitation, and microhabitats mediate space competition between coral and crustose coralline algae in a spatially explicit model. Ecological Modelling 237–238: 23–33.

[35] Melbourne-Thomas J, Johnson CR, Fung T, Seymour RM, Chérubin LM, et al (2011) Regional-scale scenario modeling for coral reefs: a decision support tool to inform management of a complex system. Ecol Appl 21: 1380–1398. 21774437 10.1890/09-1564.1

[36] Reuter H (2005) Community processes as emergent properties: Modelling multilevel interaction in small mammals communities. Ecol Modell 186: 427–446.

[37] Breckling B, Middelhoff U, Reuter H (2006) Individual-based models as tools for ecological theory and application: Understanding the emergence of organisational properties in ecological systems. Ecol Modell 194: 102–113.

[38] Grimm V, Berger U, Bastiansen F, Eliassen S, Ginot V, et al (2006) A standard protocol for describing individual-based and agent-based models. Ecol Modell 198: 115–126.

[39] Breckling B, Reuter H, Middelhoff U (1997) An object oriented modelling strategy to depict activity pattern of organisms in heterogeneous environments. Environ Model Assess 2: 95–104.

[40] Lesser MP (1997) Oxidative stress causes coral bleaching during exposure to elevated temperatures. Coral Reefs 16: 187–192.

[41] Wooldridge SA (2010) Is the coral-algae symbiosis really “mutually beneficial” for the partners? BioEssays 615–625. 20517874 10.1002/bies.200900182

[42] Ortiz JC, del C.Gomez-Cabrera M, Hoegh-Guldberg O (2009) Effect of colony size and surrounding substrate on corals experiencing a mild bleaching event on Heron Island reef flat (southern Great Barrier Reef, Australia). Coral Reefs 28: 999–1003.

[43] Box SJ, Mumby PJ (2007) Effect of macroalgal competition on growth and survival of juvenile Caribbean corals. Mar Ecol Prog Ser 342: 139–149.

[44] Idjadi JA, Karlson RH (2007) Spatial arrangement of competitors influences coexistence of reef-building corals. Ecology 88: 2449–2454. 18027746 10.1890/06-2031.1

[45] Chadwick NE, Morrow KM (2011) Coral Reefs: An Ecosystem in Transition. In: Dubinsky Z, Stambler N, editors. Coral Reefs: An Ecosystem in Transition. Dordrecht: Springer Netherlands. pp. 347–371.

[46] Yniguez AT, Mcmanus JW, DeAngelis DL (2008) Allowing macroalgae growth forms to emerge: Use of an agent-based model to understand the growth and spread of macroalgae in Florida coral reefs, with emphasis on *Halimeda tuna* . Ecol Modell 216: 60–74.

[47] Sleeman JC, Boggs GS, Radford BC, Kendrick GA (2005) Using Agent-Based Models to Aid Reef Restoration: Enhancing Coral Cover and Topographic Complexity through the Spatial Arrangement of Coral Transplants. Restoration Ecol 13: 685–694.

[48] Koehl M, Strother J, Reidenbach M, Koseff J, Hadfield M (2007) Individual-based model of larval transport to coral reefs in turbulent, wave-driven flow: behavioral responses to dissolved settlement inducer. Mar Ecol Prog Ser 335: 1–18.

[49] Brandt M, McManus J (2009) Dynamics and impact of the coral disease white plague: insights from a simulation model. Dis Aquat Org 87: 117–133. 20095247 10.3354/dao02137

[50] Tam T, Ang Jr PO (2009) Catastrophic regime shifts in coral communities exposed to physical disturbances: simulation results from object-oriented 3-dimensional coral reef model. J Theor Biol 259: 193–208. 19306887 10.1016/j.jtbi.2009.03.014

[51] McClanahan TR (2004) The relationship between bleaching and mortality of common corals. Mar Biol 144: 1239–1245.

[52] Rinkevich B, Sakai K (2001) Interspecific interactions among species of the coral genus *Porites* from Okinawa, Japan. Zoology 104: 91–97. 16351822 10.1078/0944-2006-00014

[53] Getz WM (1996) A Hypothesis Regarding the Abruptness of Density Dependence and the Growth Rate of Populations. Ecology 77: 2014–2026.

[54] Ramos-Jiliberto R, González-Olivares E (2000) Relating behavior to population dynamics: a predator – prey metaphysiological model emphasizing zooplankton diel vertical migration as an inducible response. Ecol Modell 127: 221–233.

[55] Wallace CC (1999) Staghorn corals of the world: a revision of the coral genus *Acropora*. Collingwood, Australia: Csiro Publishing. p.

[56] Hodgson (1998) Corals. In: Carpenter KE, Niem VH, editors. FAO species identifidication guide for fishery purposes. The living marine resources of the Western Central Pacific. Vol. 1. Seaweeds, coral, bivalves and gastropods. Rome: FAO. pp. 101–122.

[57] Ateweberhan M, Bruggemann JH, Breeman AM (2005) Seasonal dynamics of *Sargassum ilicifolium* (*Phaeophyta*) on a shallow reef flat in the southern Red Sea (Eritrea). Mar Ecol Prog Ser 292: 159–171.

[58] Ateweberhan M, Bruggemann JH, Breeman AM (2009) Seasonal changes in size structure of *Sargassum* and *Turbinaria* populations (*Phaeophyceae*) on tropical reef flats in the southern red sea. Journal of Phycology 45: 69–80. 27033646 10.1111/j.1529-8817.2008.00639.x

[59] Muhando CA (2002) Seawater Temperature on Shallow Reefs Off Zanzibar Town, Tanzania. In: Linden O, Souter D, Wilhelmsson D OD, editor. Coral Reef Degradation in the Indian Ocean. CORDIO Status Report 2002. pp. 40–46.

[60] Bryceson I, De Souza F, Jehanger I, Ngoile MAK, Wynter P (1990) State of the marine environment in the Eastern African region. UNEP Regional Seas Program Reports (Report # 113). Nairobi, Kenya. p.

[61] Guard M, Masaiganah M (1997) Dynamite Fishing in Southern Tanzania, Geographical Variation, Intensity of Use and Possible Solutions. Mar Pollut Bull 34: 758–762.

[62] Muhando CA, Francis J (2000) The status of coral reefs in the Dar-Es-Salaam marine reserves system and the state of reefs in other marine protected areas of Tanzania. Status Report 2000. Zanzibar: Institute for Marine Sciences/UNEP/ICLAN. 23 pp.

[63] Burkepile DE, Hay ME (2009) Nutrient versus herbivore control of macroalgal community development and coral growth on a Caribbean reef. Mar Ecol Prog Ser 389: 71–84.

[64] Birkeland C (1997) Disturbances to reefs in recent times. In: Birkeland C, editor. Life and Death of Coral Reefs. New York: Chapman and Hall. pp. 365–367.

[65] McCook LJ, Almany GR, Berumen ML, Day JC, Green a L, et al (2009) Management under uncertainty: guide-lines for incorporating connectivity into the protection of coral reefs. Coral Reefs 28: 353–366.

[66] Nyström M (2006) Redundancy and Response Diversity of Functional Groups: Implications for the Resilience of Coral Reefs. Ambio 35: 30–35. 16615697

[67] Bjork M, Mohammad SM, Bjorklund M, Semesi A (1995) Coralline algae, important coral reef builders threatened by pollution. AMBIO 24: 502–505.

[68] DeGeorges A, Goreau TJ, Reilly B (2010) Land-Sourced Pollution with an Emphasis on Domestic Sewage: Lessons from the Caribbean and Implications for Coastal Development on Indian Ocean and Pacific Coral Reefs. Sustainability 2: 2919–2949.

[69] Rushingisha G (2012) Modeling coral-corallimorpharia interaction under varying anthropogenic inputs along the coast of Tanzania [M.Sc. thesis].Zanzibar (Tanzania) : Institute for Marine Sciences, University of Dar es Salaam. 140 pp.

[70] Muhando Ca, Kuguru BL, Wagner GM, Mbije NE, Ohman MC (2002) Environmental effects on the distribution of corallimorpharians in Tanzania. Ambio 31: 558–561. 12572822

[71] Muhando CA, Lanshammar F (2008) Ecological Effects of the Crown-of-Thorns Starfish Removal Programme on Chumbe Island Coral Park, Zanzibar, Tanzania. Proceedings of the 11th International Coral Reef Symposium 7–11.

[72] Hughes TP, Connell JH (1999) Multiple stressors on coral reefs: A long-term perspective. Limnol Oceanogr 44: 932–940.

[73] Côté IM, Darling ES (2010) Rethinking ecosystem resilience in the face of climate change. PLoS Biology 8: e1000438. 20668536 10.1371/journal.pbio.1000438 PMC2910654

[74] Rachello-Dolmen PG, Cleary DFR (2007) Relating coral species traits to environmental conditions in the Jakarta Bay/Pulau Seribu reef system, Indonesia. Estuarine, Coastal and Shelf Science 73: 816–826.

[75] Grime JP (1973) Competitive Exclusion in Herbaceous Vegetation. Nature 242: 344–347.

[76] Luckhurst BE, Luckhurst K (1978) Analysis of influence of substrate variables on coral-reef fish communities. Mar Biol 49: 317–323.

[77] Hixon MA, Beets JP (1993) redation, Prey Refuges, and the Structure of Coral-Reef Fish Assemblages. Ecol Monogr 63: 77–101.

[78] Kuffner I, Walters L, Becerro M, Paul V, Ritson-Williams R, et al (2006) Inhibition of coral recruitment by macroalgae and cyanobacteria. Mar Ecol Prog Ser 323: 107–117.

[79] Hoey AS, Pratchett MS, Cvitanovic C (2011) High macroalgal cover and low coral recruitment undermines the potential resilience of the world's southernmost coral reef assemblages. PLoS ONE 6: e25824. 21991366 10.1371/journal.pone.0025824 PMC3185058

[80] McClanahan T, Ateweberhan M, Graham N, Wilson S, Sebastián C, et al (2007) Western Indian Ocean coral communities: bleaching responses and susceptibility to extinction. Mar Ecol Prog Ser 337: 1–13.

[81] McClanahan TR, Muthiga NA, Mangi S (2001) Coral and algal changes after the 1998 coral bleaching: interaction with reef management and herbivores on Kenyan reefs. Coral Reefs 19: 380–391.

[82] Scheffer M, van Nes EH, Holmgren M, Hughes T (2008) Pulse-Driven Loss of Top-Down Control: The Critical-Rate Hypothesis. Ecosystems 11: 226–237.

[83] Hughes TP, Rodrigues MJ, Bellwood DR, Ceccarelli D, Hoegh-Guldberg O, et al (2007) Phase Shifts, Herbivory, and the Resilience of Coral Reefs to Climate Change. Current Biology 17: 360–365. 17291763 10.1016/j.cub.2006.12.049

[84] Obura D, Grimsditch G (2009) Coral Reefs, Climate Change and Resilience. In: Obura D, Grimsditch G, editors. An Agenda for Action from the IUCN World Conservation Congress in Barcelona, Spain. Gland, Switzerland: IUCN. p. 44.

[85] Mumby PJ, Steneck RS (2011) The Resilience of Coral Reefs and its Implications for Reef Management. In: Dubisky Z, Stambler N, editors. Coral Reefs: An Ecosystem in Transition. Springer Science+Business Media B.V. pp. 509–520.

[86] Edinger E (2000) Normal Coral Growth Rates on Dying Reefs: Are Coral Growth Rates Good Indicators of Reef Health? Mar Pollut Bull 40: 404–425.

[87] Guzman HM, Cortes J (1989) Growth rates of eight species of scleractinian corals in the Eastern Pacific (Costa Rica). Bulletin of Marine Science 44: 1186–1194.

[88] Grigg RW (2006) Depth limit for reef building corals in the Au'au Channel, S.E. Hawaii. Coral Reefs 25: 77–84 doi:10.1007/s00338-005-0073-6.

[89] de Villiers S, Shen GT, Nelson BK (1994) The Sr/Ca-temperature relationship in coralline aragonite: Influence of variability in (Sr/Ca). Science 58: 197–208.

[90] Grottoli AG (1999) Variability of stable isotopes and maximum linear extension in reef-coral skeletons at Kaneohe Bay, Hawaii. Mar Biol 135: 437–449.

[91] Smith LW, Barshis D, Birkeland C (2007) Phenotypic plasticity for skeletal growth, density and calcification of *Porites lobata* in response to habitat type. Coral Reefs 26: 559–567.

[92] Floral CJ, Ely PS (2003) Surface Growth Rings of *Porites lutea* Microatolls Accurately Track Their Annual Growth. Nonhwest Science 77..

[93] Rosenfeld M, Yam R, Shemesh A, Loya Y (2003) Implication of water depth on stable isotope composition and skeletal density banding patterns in a *Porites lutea* colony: results from a long-term translocation experiment. Coral Reefs 22: 337–345.

[94] Highsmith RC (1979) Coral growth-rates and environmental control of density banding. J Exp Mar Biol Ecol 37: 105–125.

[95] Tanzil JTI, Brown BE, Tudhope a W, Dunne RP (2009) Decline in skeletal growth of the coral *Porites lutea* from the Andaman Sea, South Thailand between 1984 and 2005. Coral Reefs 28: 519–528.

[96] Supriharyono (2004) Growth rates of the massive coral *Porites lutea* Edward and Haime, on the coast of Bontang, East Kalimantan, Indonesia. Journal of Coastal Development 7: 143–155.

[97] Bessat F, Buigues D (2001) Two centuries of variation in coral growth in a massive *Porites* colony from Moorea (French Polynesia): a response of ocean-atmosphere variability from south central Pacific. Palaeogeography, Palaeoclimatology, Palaeoecology 175: 381–392.

[98] Suzuki A, Hibino K, Iwase A, Kawahata H (2005) Intercolony variability of skeletal oxygen and carbon isotope signatures of cultured *Porites* corals: Temperature-controlled experiments. Geochimica et Cosmochimica Acta 69: 4453–4462.

[99] Oliver JK, Chalker BE, Dunlap WC (1983) Bathymetric adaptations of reef-building corals at Davies Reef, Great Barrier Reef, Australia. I. Long-term growth responses of *Acropora formosa* (Dana 1846). J Exp Mar Biol Ecol 73: 11–35.

[100] Jinendradasa SS, Ekaratne SUK (2000) Linear extension of *Acropora formosa* (Dana) at selected reef locations in Sri Lanka. In: Proceedings of the 9th International Coral Reef Symposium; 23–27 October 2000; Bali, Indonesia. ICRS 2000.

[101] Harriott VJ (1998) Growth of the staghorn coral *Acropora formosa* at Houtman Abrolhos, Western Australia. Mar Biol 132: 319–325.

[102] Oliver JK (1984) Intra-colony Variation in the Growth of *Acropora formosa*: Extension Rates and Skeletal Structure of White (Zooxanthellae-free) and Brown-Tipped Branches. Coral Reefs 3: 139–147.

[103] Dennison WC, Barnes DJ (1988) Effect of water motion on coral photosynthesis calcification. Water 115: 67–71.

[104] Charuchinda M, Hylleberg J (1984) Skeletal Extension of *Acropora formosa* at a Fringing Reef in the Andaman Sea. Coral Reefs 3: 215–219.

[105] Wellington GM (1982) An Experimental Analysis of the Effects of Light and Zooplankton on Coral Zonation. Oecologia 311–320. 10.1007/BF00367953 28310389

[106] Neudecker S (1981) Growth and Survival of Scleractinian Corals exposed to thermal Effluents at Guam. In: Proceedings of the 4th International Coral Reef Symposium; 18–22 May 1981; Manila, Philippines. ICRS 1981.

[107] Harriott VJ (1999) Coral growth in subtropical eastern Australia. Coral Reefs 18: 281–291.

[108] Weil SM, Buddemeier RW, Smith SV, Kroopnick PM (1981) The stable isotopic composition of coral skeletons: control by environmental variables. Geochimica et Cosmochimica Acta 45: 1147–1153.

[109] Romano S (1990) Long-term effects of interspecific aggression on growth of the reef-building corals *Cyphastrea ocellina* (Dana) and *Pocillopora damicornis* (Linnaeus). J Exp Mar Biol Ecol 140: 135–146.

[110] Glynn PW, Stewart RH (1973) Distribution of Coral Reefs in the Pearl Islands (Gulf of Panama) in Relation to thermal conditions. Limnology and Oceanography 18: 367–379.

[111] Le Tissier MDA (1988) The growth and formation of branch tips of *Pocillopora damicornis* (Linnaeus). J Exp Mar Biol Ecol 124: 115–131.

[112] Ward S (1995) The effect of damage on the growth, reproduction and storage of lipids in the scleractinian coral *Pocillopora damicornis* (Linnaeus). J Exp Mar Biol Ecol 187: 193–206.

[113] Clark TH (1998) The ecology of indigenous and transplanted corals in the Cape d'Aguilar Marine Reserve, Hong Kong [PhD dissertation]. University of Hong Kong. 618. pp. http://hub.hku.hk/handle/10722/31678

[114] Harriott VJ (1983) Reproductive Seasonality, Settlement, and Post-settlement Mortality of *Pocillopora damicornis* (Linnaeus), at Lizard Island, Great Barrier Reef. Coral Reefs 151–157.

[115] Harrison PL, Wallace CC (1990) Reproduction, dispersal and recruitment of scleractinian corals. In: Dubinsky Z, editor. Ecoystems of the World. New York and Amsterdam: Elsevier Science. pp. 133–207.

[116] Mangubhai S (2007) Reproduction and recruitment of scleractinian corals on equatorial reefs in Mombasa, Kenya. Reproduction

[117] Manusik, Suharsono, Situmorang J, Kamiso HN (2008) Timing of Larval Release by Reef Coral *Pocillopora damicornis* at Panjang Island, Central Java. Mar Res Indonesia 33: 33–39.

[118] Okubo RTN, Taniguchi H, Motokawa T (2005) Successful methods for transplanting fragments of *Acropora formosa* and *Acropora hyacinthus* . Coral Reefs 24: 333–342.

[119] Rinkevich B, Loya Y (1979) The Reproduction of the Red Sea Coral *Stylophora pistillata*. I. Gonads and Planulae. Mar Ecol Prog Ser 1: 133–144.

[120] Veron, J. E. N., Stafford-Smith, Mary, Australian Institute of Marine Science (2000) *Corals of the world*. Townsville, Qld: Australian Institute of Marine Science.

[121] Penland L, Kloulechad J, Idip D, van Woesik R (2004) Coral spawning in the western Pacific Ocean is related to solar insolation: evidence of multiple spawning events in Palau. Coral Reefs 23: 133–140.

[122] Richmond RH, Hunter CL (1990) Reproduction and recruitment of corals: comparisons among the Caribbean, the Tropical Pacific, and the Red Sea. Mar Ecol Prog Ser 60: 185–203.

[123] Babcock RC, Bull GD, Harrison PL, Heyward AJ, Oliver JK, et al (1986) Synchronous spawnings of 105 scleractinian coral species on the Great Barrier Reef. Mar Biol 90: 379–394.

[124] Ward S (1992) Evidence for broadcast spawning as well as brooding in the scleractinian coral *Pocillopora damicornis* . Mar Biol 112: 641–646.

