# Supplementary figures and images for: Simulations of Long-Term Community Dynamics in Coral Reefs - How Perturbations Shape Trajectories
Source: PLoS Comput Biol. 2012 Nov 29;8(11):e1002791. doi: 10.1371/journal.pcbi.1002791 (PMC3510096; doi:10.1371/journal.pcbi.1002791)

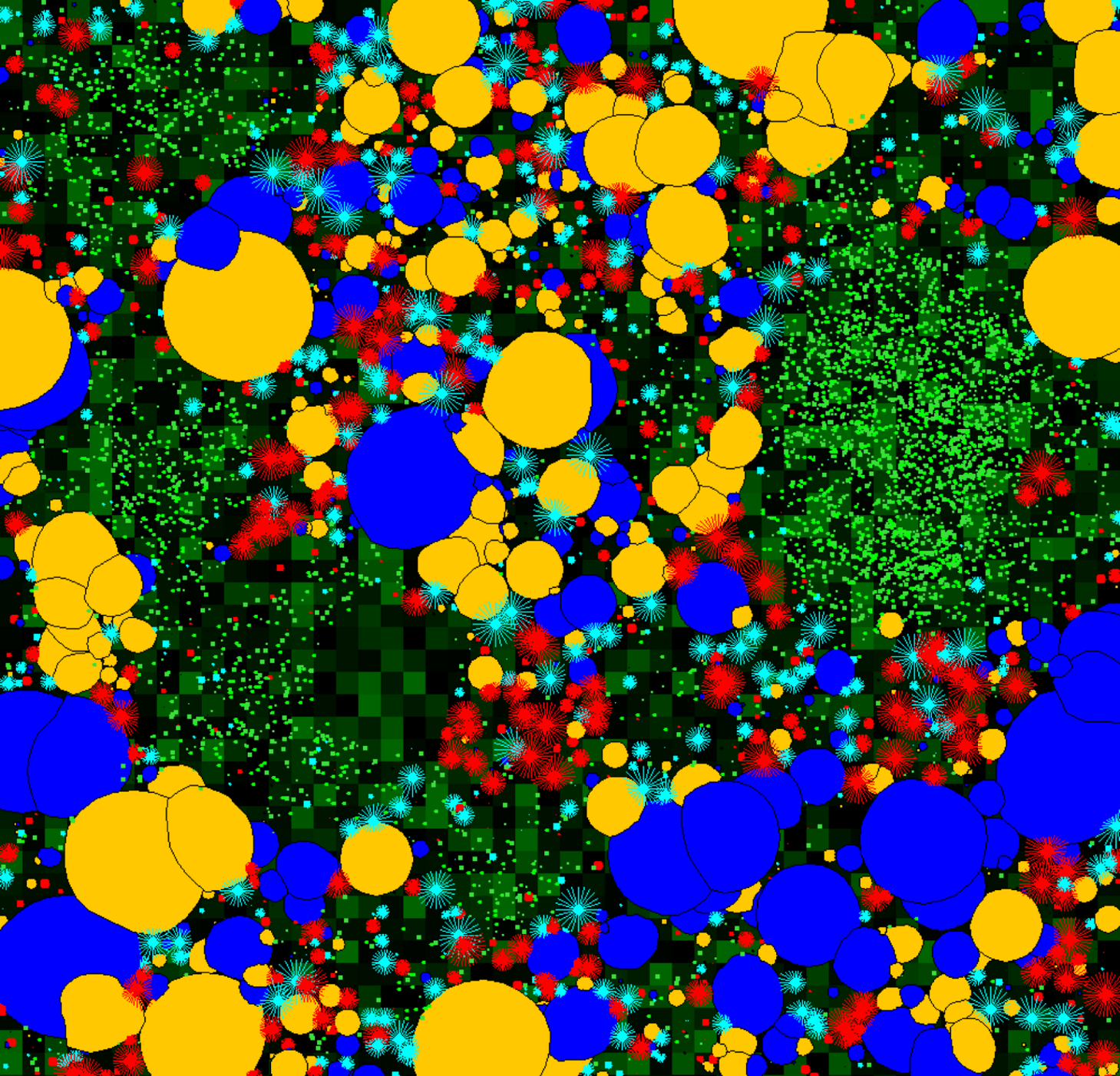

Supplement: Figure S1 — Screen shot of the graphical user interface during a simulation. Colonies of the two massive species P. lobata (blue polygons) and P. lutea (orange polygons), and the two branching species A. muricata (red stars) and P. damicornis (cyan stars) compete for space with each other as well as with macroalgae (green dots) and algal turf (green squares). The free space which is largely covered by macroalgae and turf indicates post-disturbed areas. (TIF) [file pcbi.1002791.s001.tif]

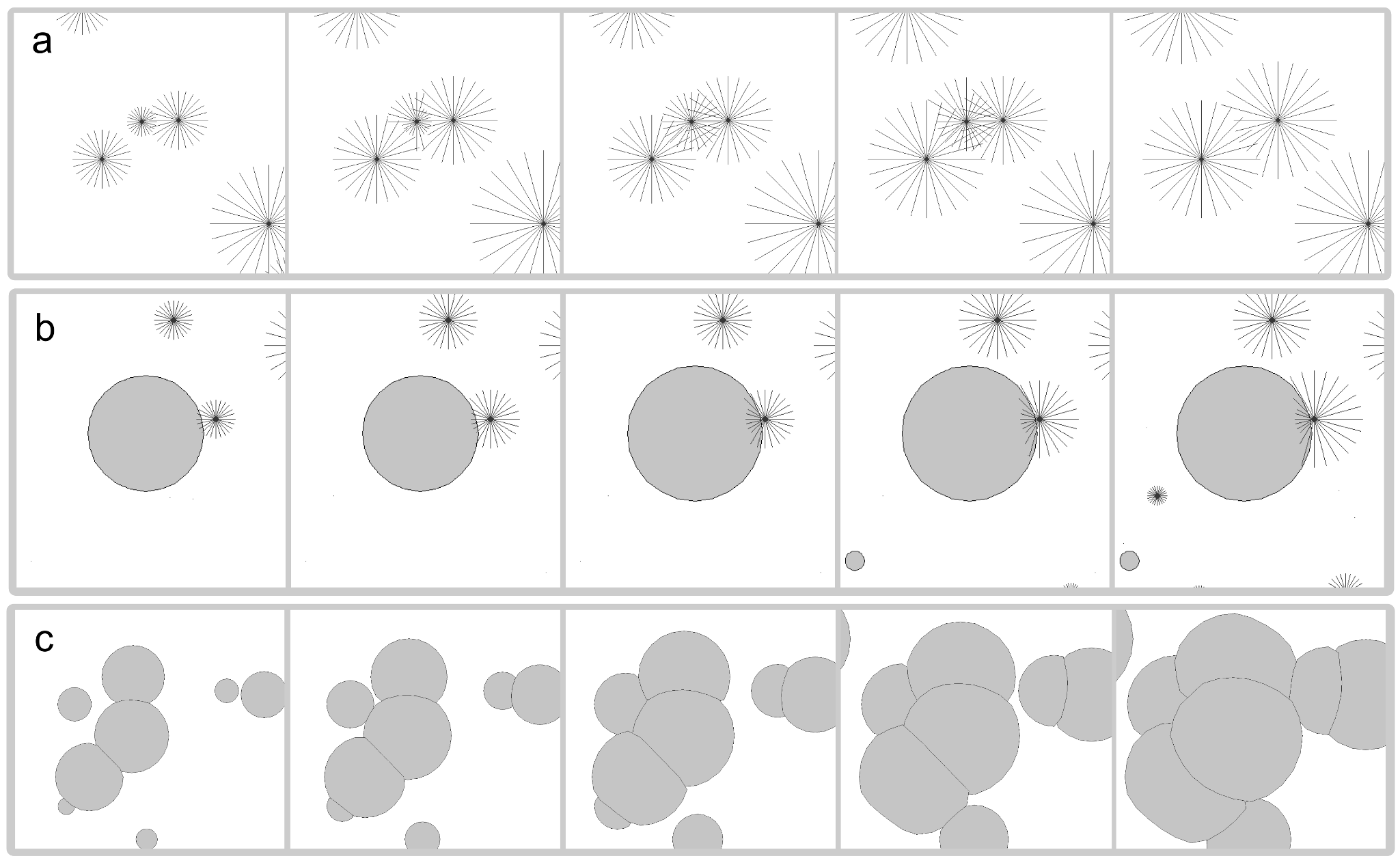

Supplement: Figure S2 — Interaction between different neighboring coral colonies. Different growth forms interact in different ways which is shown for (a) branching colonies, (b) massive with branching colonies and (c) massive colonies, respectively. Growth is clearly restricted in direction of neighboring individuals and thus the common irregular shapes arise. (TIF) [file pcbi.1002791.s002.tif]

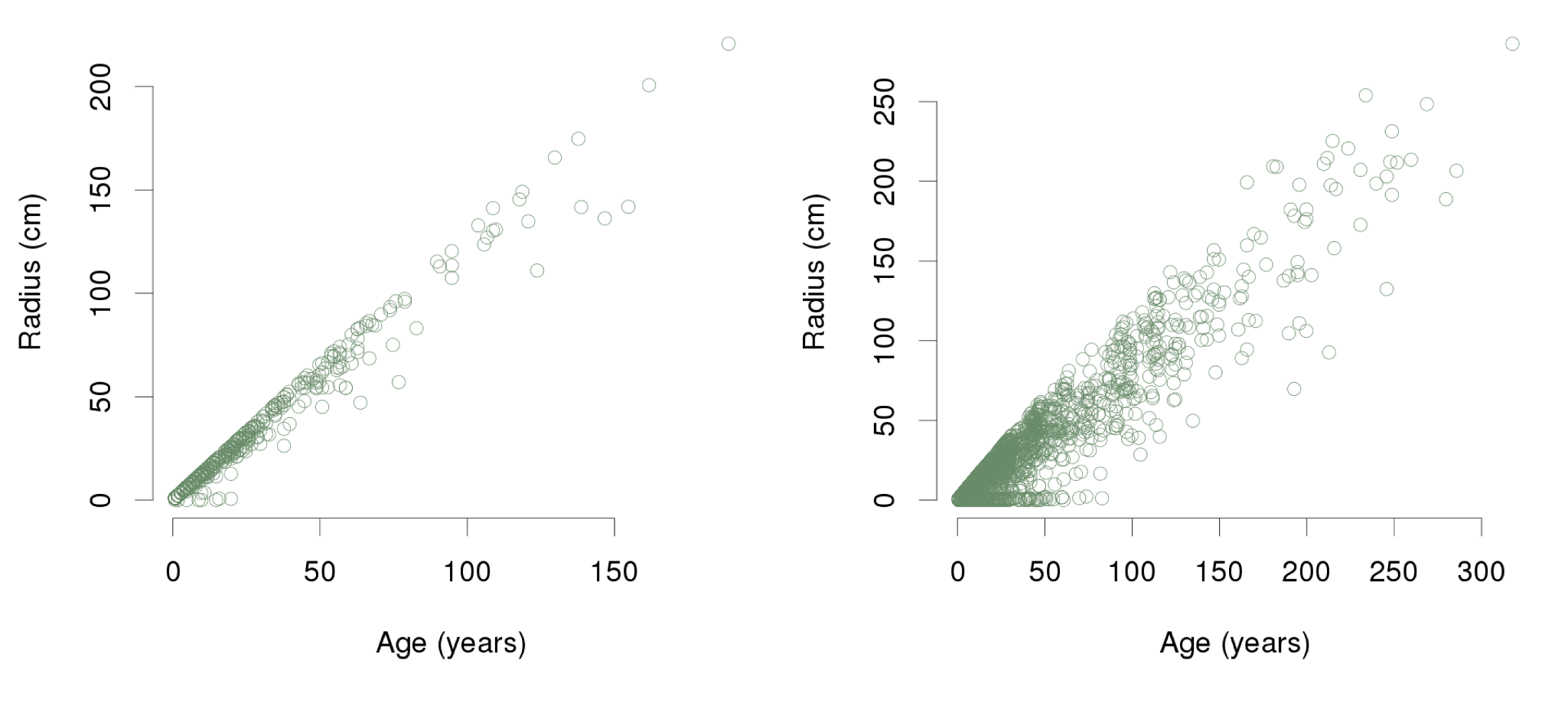

Supplement: Figure S3 — The population's growth performance of Porites lutea under different disturbance levels and crowding regimes. high disturbance levels, which imply low crowding (left), and low disturbance levels which imply high crowding (right), respectively. (TIF) [file pcbi.1002791.s003.tif]

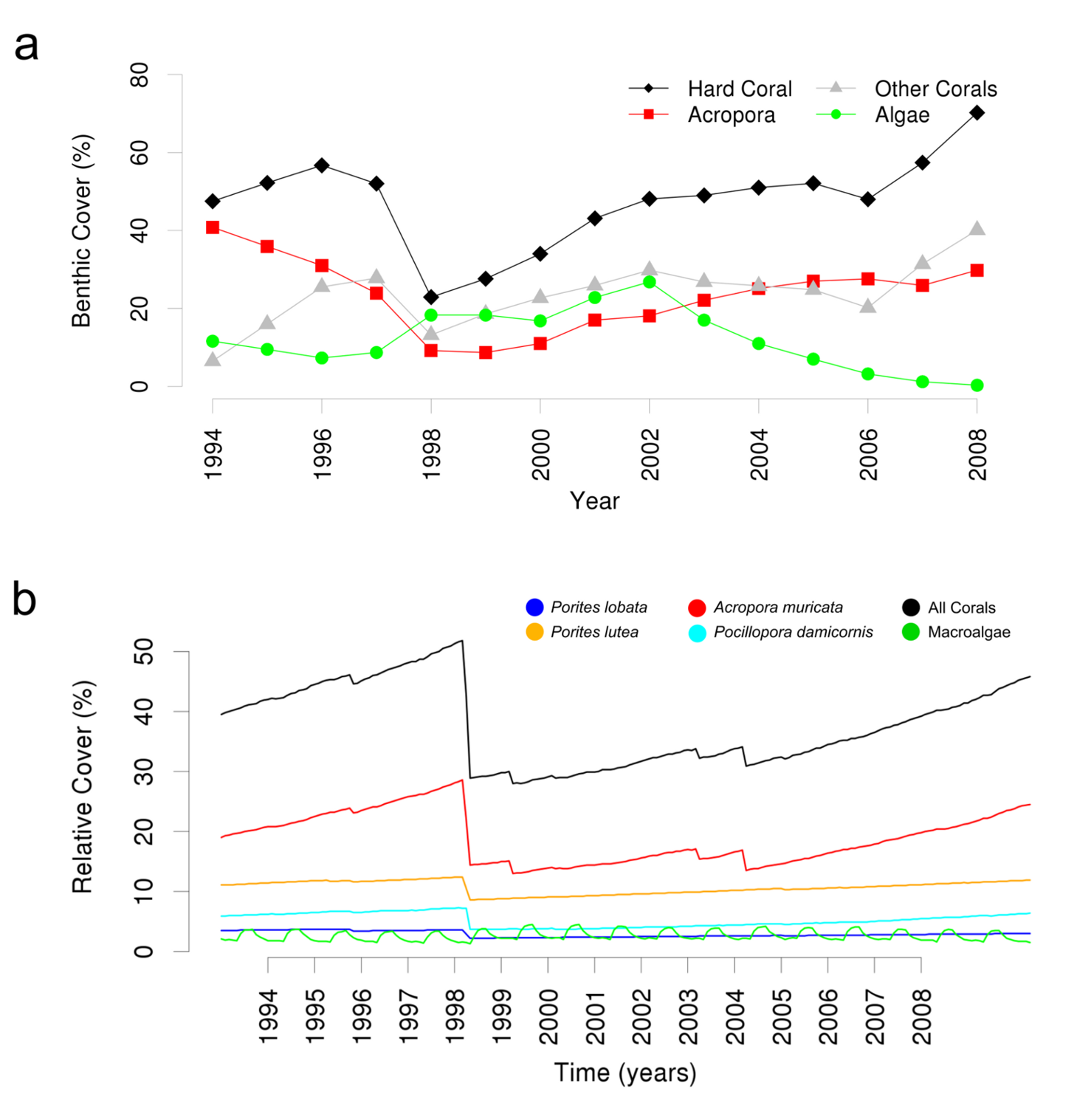

Supplement: Figure S4 — Validation of the community response to a major bleaching event. In (a) the relative benthic cover of Chumbe Island MPA is shown before and after the 1998 bleaching event (adapted from Muthiga et al. [7] in Text S1). Chart (b) shows a time line of the model output that represents the impact of the 1998 bleaching event. (TIF) [file pcbi.1002791.s004.tif]

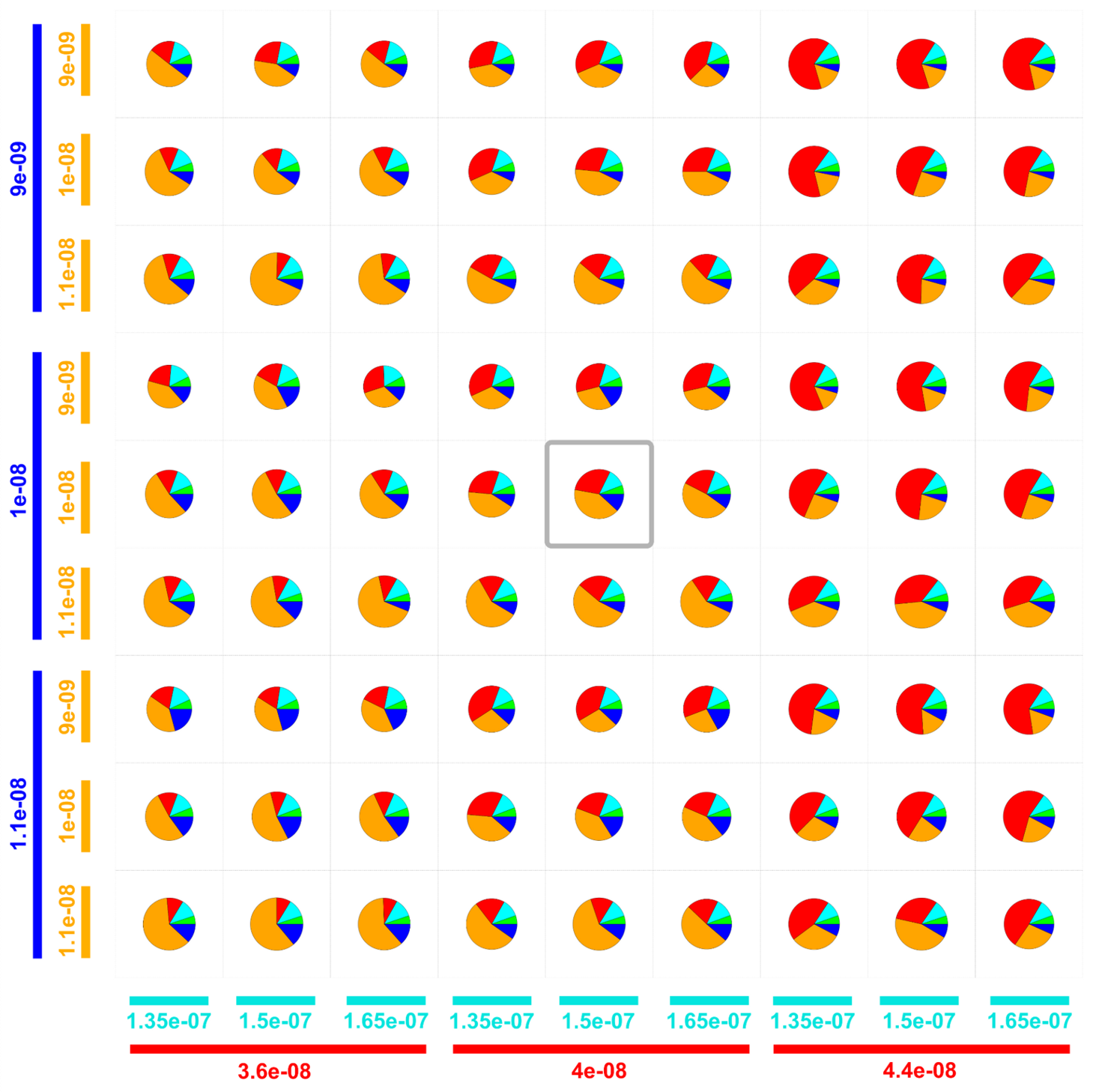

Supplement: Figure S5 — Retention factors of all coral species were varied by ±10% of their standard value and respective community responses plotted for each setting. Vertically the retention rates of the two massive species (P. lobata in blue and P. lutea in orange) are varied and horizontally the ones of the two branching species (A. muricata in red and P. damicornis in cyan). The size of the pie chart indicates the total benthic cover and the gray box indicates the standard values. (TIF) [file pcbi.1002791.s005.tif]

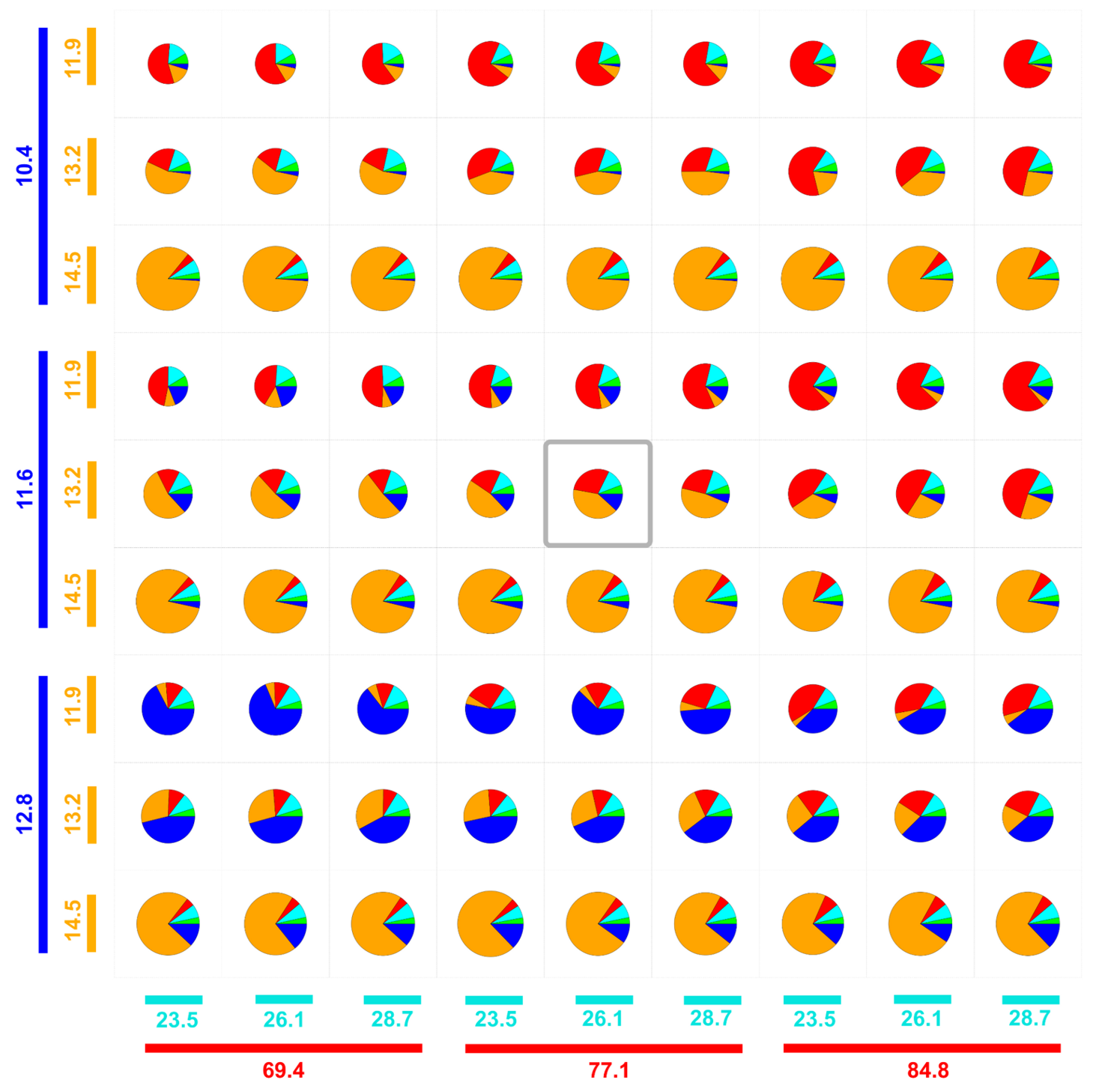

Supplement: Figure S6 — Growth rates of all coral species were varied by ±10% of their standard value and respective community responses plotted for each setting. Vertically the growth rates of the two massive species (P. lobata in blue and P. lutea in orange) are varied and horizontally the ones of the two branching species (A. muricata in red and P. damicornis in cyan). The gray box indicates the standard values. (TIF) [file pcbi.1002791.s006.tif]

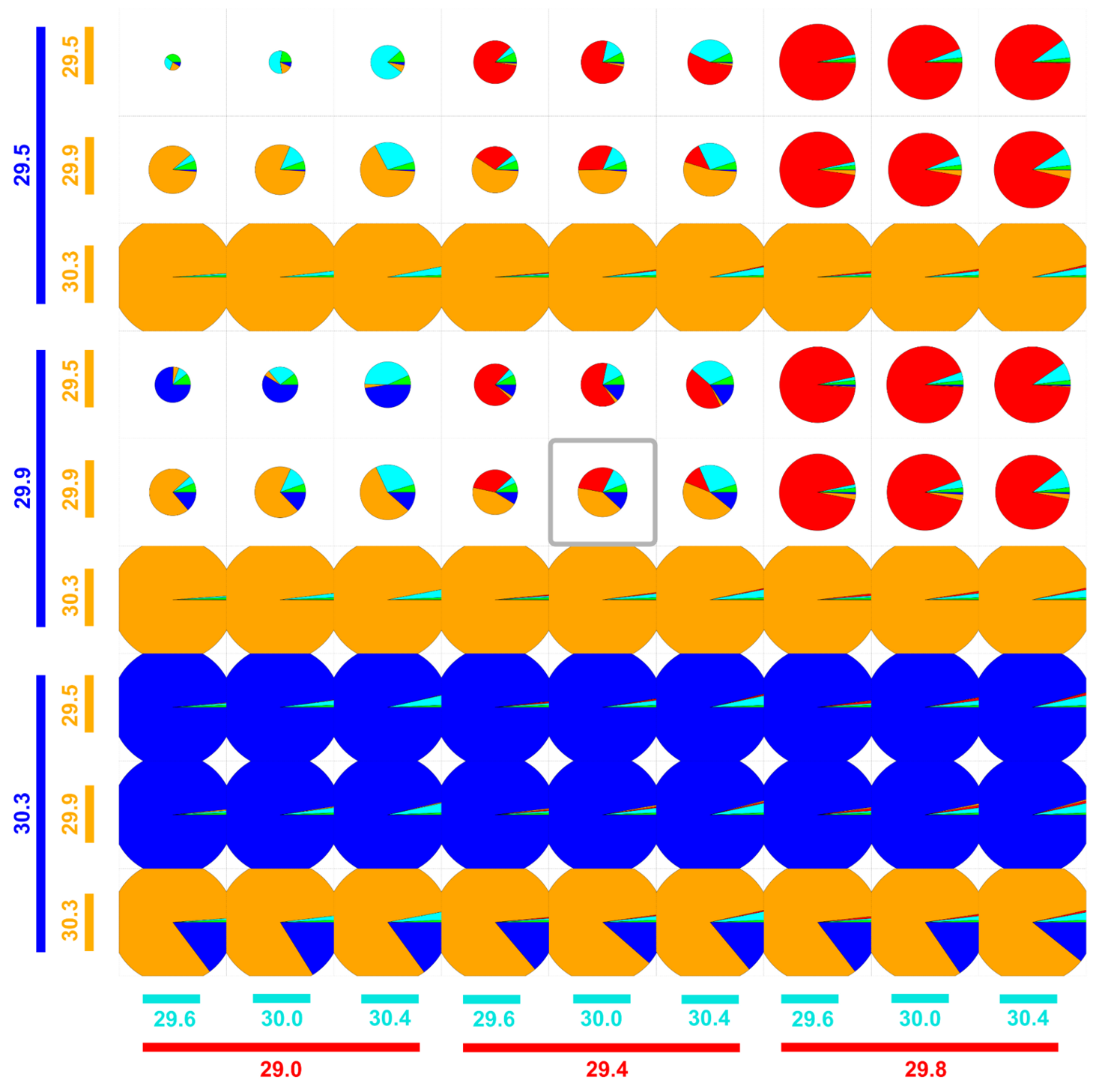

Supplement: Figure S7 — The minimum bleaching temperature for each coral species was varied by ±0.4°C of its respective standard value and the community response was plotted for each setting. Vertically the growth rates of the two massive species (P. lobata in blue and P. lutea in orange) are varied and horizontally the ones of the two branching species (A. muricata in red and P. damicornis in cyan). The size of the pie chart indicates the total benthic cover and the gray box indicates the standard values. (TIF) [file pcbi.1002791.s007.tif]

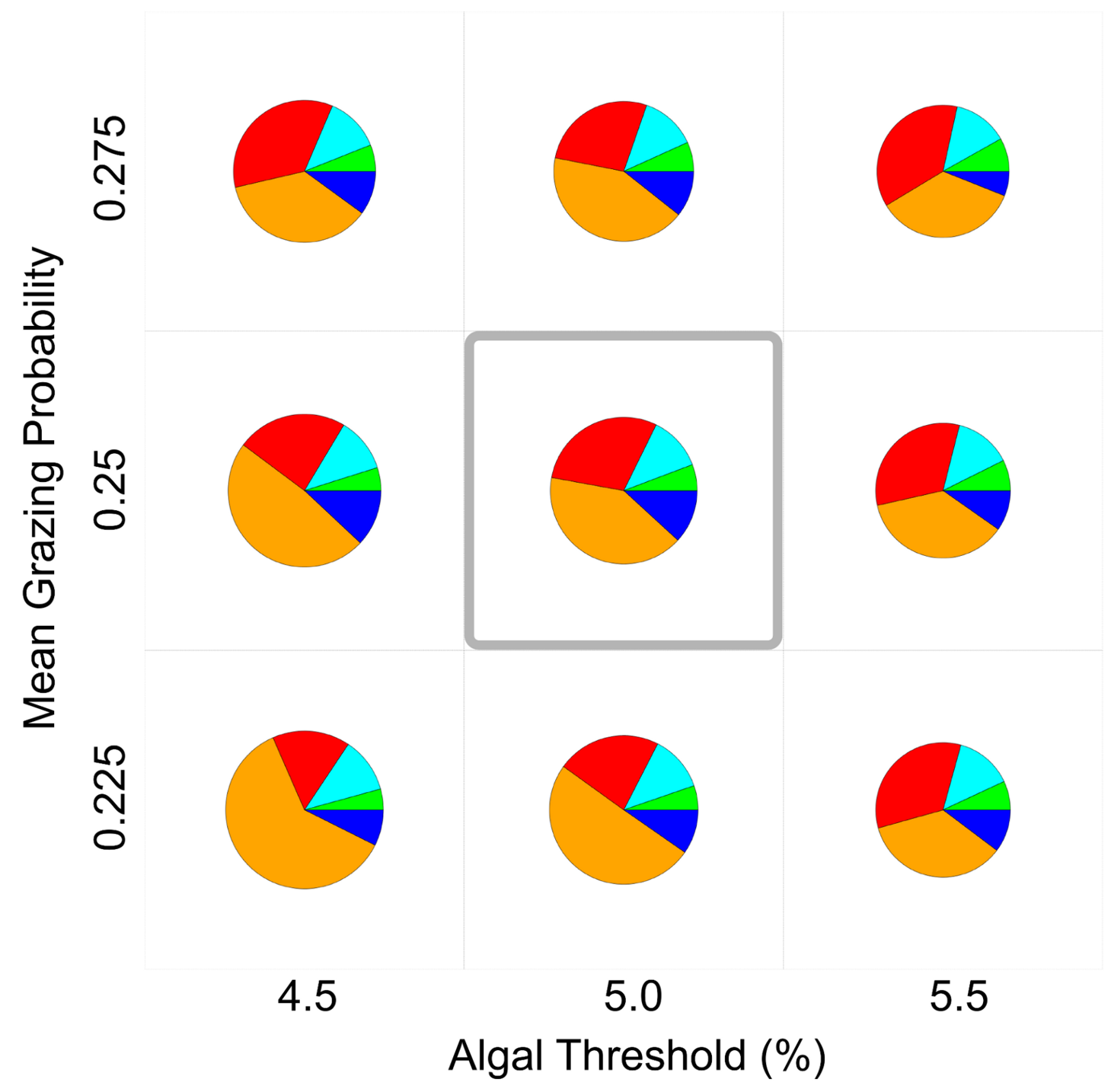

Supplement: Figure S8 — Variation of key parameters for herbivory (±10%) shows no decisive effect on coverage and community composition. The colours represent benthic organisms as follows: P. lobata in blue, P. lutea in orange, A.muricata in red, P. damicronis in cyan and macroalgae in green. (TIF) [file pcbi.1002791.s008.tif]
