# Supplementary material for: Simulations of Long-Term Community Dynamics in Coral Reefs - How Perturbations Shape Trajectories
Source: PLoS Comput Biol. 2012 Nov 29;8(11):e1002791. doi: 10.1371/journal.pcbi.1002791 (PMC3510096; doi:10.1371/journal.pcbi.1002791)
Supplement: Text S1 — Appendix with a detailed sensitivity analysis and a hierarchically structured validation for the model. (PDF) [file pcbi.1002791.s009.pdf]

Simulations of Long-Term Community Dynamics in Coral Reefs
– How Perturbations Shape Trajectories –

A. Kubicek, C. Muhando, H. Reuter

Sensitivity analysis and validation

In order to ensure correctness of model properties and provide information on model reliability, we applied different techniques which may be subsumed under the broad and overlapping term of 'validation':

1. The comparison of model processes, dynamics and results with available data and current knowledge on the reef dynamics
2. A sensitivity analysis to investigate which parameters, or combinations of parameters lead to relevant changes in model outcomes.

These techniques aim at specific aspects of model correctness which will be elaborated in the following sections. For individual-based models calibration and parameter fitting are comparatively less demanding as the applied parameters are very near to biological processes and empirical data, which leads to a narrow and clearly definable specific range of plausible values [1].

Model comparison with field data and observations

IBM requires a specific approach for validation [1] as they represent complex ecological situations, often with a large number of components and many varying relationships. In this situation a standard statistical approach [2] to model validation by simply comparing model results with a specific data set is not meaningful enough.

The detailed level of representation of organismic processes in IBMs allows for a different approach of model validation which is followed here. The accuracy of model processes and outcomes is checked at different integration levels comprising the range from individual life-histories to community dynamics. Here a 'hierarchically structured validation' [3] investigates to what extent model mechanisms reproduce the proposed characteristics of the studied ecological context that is known from expert knowledge and that is described by the conceptual model. In this case the model should not only reproduce the observed system dynamics on different levels but should also reflect the processes and causal level which generates the behavior in the real system [4]. As model dynamics result from self-organization processes a correct representation of key processes on lower hierarchical organization levels increases the probability that (i) the system behavior has been represented correctly, and (ii) that the results are trustworthy within the specified conditions and the implemented conceptual system.

Individual level

The individual level is very close to the implemented model processes. The main task on this level is to check the accuracy of implementation and the consistency of sequence in life-cycles and with current knowledge. In our analysis on this level we focus on the development of individual corals and

their interaction with other corals. The reaction to neighbors is an important process in a model which emphasizes the representation of spatial interaction and competition. The outcome of the competition depends on factors, like local density, the individual growth rate and rules which determine interaction (see also section 2.1 c). As defined by the rules, growth is clearly restricted in direction of neighboring individuals and common irregular shapes arise (Fig. S2).

Population level

The population level constitutes an important integration level to control and evaluate the implementation and the effects of individual parameters. The results on this level are based on the interactions of the individuals in their respective interaction ranges. The overall results on this level thus emerge as a self-organized process. As an example of how individual interactions produce plausible population properties, we show altering growth rates upon interactions among neighboring organisms.

Coral growth has been described as linear extension rates which are influenced by neighboring corals and by the state of each coral. Thus it can be expected that the age-dependent size distribution (Fig. S3) of a coral population differs clearly from the linear relationship which we would get without any influences. Furthermore it should vary according to the different development phases of the population, the overall community density and external influences.

Community level

In principle on this level the same conditions apply as on the population level, but with an additional consideration of inter-species interactions.

During the 1998 major bleaching event in the Western Indian Ocean region many reefs showed tremendous declines in coral cover. At Chumbe Island reserve the total hard coral cover decreased from >50% to about 20-25% (Fig. S4 a) which was also confirmed by Muhando and Mohammad [5]. In particular, the cover of *Acropora* species decreased from about 25 to 10%. The model, which was parameterized with bleaching data of the 1998 bleaching event from Kenya [6] represents these characteristics quite well (Fig. S4 b). Here the total coral cover is reduced from >50% to ~30% and that of *A. muricata* from about 25% to 10%. The regrowth of the reef seems to be quicker in the Chumbe reef site which might be explained by a higher variety of coral species, of which several may exhibit higher growth rates than those represented in the model.

Sensitivity analysis

The sensitivity analysis provides information on how specific parameter values influence model results. In this way it can be determined whether parameters with a potentially high uncertainty have a critical influence on the model output. Those parameters, which cause large effects on overall results upon small variations of values should receive high attention during parameterization with the aim of minimizing uncertainty.

For our analysis we concentrated on parameters which related directly to properties of the coral species, and were identified during model development to alter the model's behavior tremendously upon small changes of values. Among these parameters we tested growth and reproduction of corals, the bleaching reaction to temperature, and the impact of herbivory on coral abundances, and varied these parameters in a biologically plausible range:

- Growth rates as well as larval retention factors for all coral species were varied by ± 10% of their standard value and tested in all possible combinations to estimate their influence on the

population's growth performance, relative abundance, and population age structure. This approach allows analyzing inherent model properties and potential reactions to absolute and relative changes in parameters.

- The temperature threshold at which a coral starts to bleach was varied for each species within a range of ± 0.4 °C. This might have a strong impact on the community composition of the observed system, as soon as qualitative differences between species arise. We chose 0.4 °C because the threshold temperature for *A. muricata* is just that much higher than the long term average temperature from which the temperature sums are calculated (Tab. 3).
- In the equation for herbivory, the input grazing probability and the algal threshold are variables which were estimated, and the influence of their variation therefore needs special attention; both parameters were changed by ± 10% and tested in all possible combinations.

Standard configuration

In the standard configuration, where major bleaching events occurred every 15 years, and small and large mechanical disturbances occurred yearly and every 5 years, respectively, the total benthic cover amounted to ~ 44% and the community was partitioned as follows: *P. lutea* made up the largest fraction with 41%, followed by *A. muricata* with 29%, the two other coral species had each a 12% share, and algae ~ 6%.

Retention factors

Varying retention rates produced highest rates of change for the dominant species in each growth morphology group, namely *P. lutea* and *A. muricata* (Fig. S5). At highest levels, *A. muricata* almost always dominated the community. *P. lutea* dominated in all other cases if its retention rate was at intermediate or highest levels. In contrast to all other sensitivity analyzes the total benthic cover was affected little by changing configurations of specific retention rates.

The retention of coral larvae is a critical parameter for the outcome of the model and, hence, has to be determined with great care.

Growth rates

The variation of growth rates had an effect on the overall benthic cover and on the community structure (Fig. S6). If *P. lutea* grew at its maximum level it always clearly dominated the community. At the highest applied growth rate *P. lobata* dominated, if *P. lutea* was growing at lowest or intermediate levels. *A. muricata* only dominated if both of the massive species were at their lowest or intermediate levels and with increasing covered area the variance also increased. *P. damicornis* was affected the least by varying growth rates.

The model reacted sensitively to growth rate alterations if qualitative changes arose (i.e. species A, which normally grew slower than species B, grew faster than species B after an alteration); and mainly within populations of the massive coral species. Therefore, we suggest that coral growth rates should be accurately determined for the investigated site.

Bleaching temperature threshold

The variation of minimum bleaching temperatures of the two massive species caused the highest alterations in overall benthic cover (Fig. S7). Low sensitivity levels for *P. lutea* generally implied the highest overall benthic cover, and *P. lutea* always held the largest fraction of the community (> 50%). On the highest tested value of the minimum bleaching temperature *P. lobata* showed similar but not as

pronounced effects on the overall outcome. If the bleaching susceptibility of *P. lutea* was at its highest or intermediate and that of *P. lobata* was at lowest levels, the overall benthic cover increased and was dominated by *P. lobata*. Analogously to the sensitivity tests for growth rates, massive corals were only influenced by branching corals at intermediate and highest bleaching sensitivities.

Within the branching coral species, *P. damicornis* dominated only in scenarios where it was least or intermediately susceptible and all other species at their most susceptible. In these cases the total benthic cover was lowest.

A. muricata disappeared completely from all simulations if its minimum bleaching temperature was set directly to the long term mean temperature, but dominated at lowest susceptibility if massive corals were at their highest and standard levels.

The reaction to bleaching reveals that small changes in susceptibilities to extreme temperatures lead to large changes in species composition and overall benthic cover. The parameterization of respective bleaching properties is very sensitive, has to be done with great care, and is of particular relevance for any alterations of temperature. We emphasize the need for more detailed studies to be conducted to reduce uncertainty in this point because specific bleaching susceptibilities are a key parameter for reef resilience and can influence reef fate at particular sites, decisively.

Herbivory impact

A change in the herbivory parameters led to surprisingly small changes in on overall model outcomes (Fig. S8). Algal cover increases slightly with an increase of the algal threshold and the total benthic cover stays approximately the same over all treatments.

References

1. Reuter H, Jopp F, Breckling B, Lange C, Weigmann G (2011) How Valid Are Model Results? Assumptions, Validity Range and Documentation. In: Jopp F, Reuter H, Breckling B, editors. Modelling Complex Ecological Dynamics. Springer Berlin Heidelberg, Vol. 9. pp. 323–340.

2. Power M (1993) The predictive validation of ecological and environmental models. Ecological Modelling 68: 33–50.

3. Reuter H, Kubicek A, Jopp F, Breckling B, Lange C (n.d.) Hierarchically Structured Validation of Individual-based Models: How to Consider Qualitative, Compositional and Quantitative Implications.

4. Troitzsch KG (2004) Validating simulation models. In: Horton G, editor. Proceedings of the 18th European Simulation Multiconference. SCS Europe. p. 6.

5. Muhando CA, Mohammed MS (2002) Coral Reef Benthos and Fisheries in Tanzania Before and After the 1998 Bleaching and Mortality Event. Western Indian Ocean Journal of Marine Science 1: 43–52.

6. McClanahan TR (2004) The relationship between bleaching and mortality of common corals. Mar Biol 144: 1239–1245.

7. Muthiga N, Costa A, Motta H, Muhando C, Mwaipopo R, et al. (2008) Status of Coral Reefs in East Africa: Kenya, Tanzania, Mozambique and South Africa. In: Wilkinson C, editor. Status of Coral Reefs of the World: 2008. Townsville, Australia: Global Coral Reef Monitoring Network and Reef and Rainforest Research Center. pp. 91–104.
